# Midkine activation of CD8^+^ T cells establishes a neuron–immune–cancer axis responsible for low-grade glioma growth

**DOI:** 10.1038/s41467-020-15770-3

**Published:** 2020-05-01

**Authors:** Xiaofan Guo, Yuan Pan, Min Xiong, Shilpa Sanapala, Corina Anastasaki, Olivia Cobb, Sonika Dahiya, David H. Gutmann

**Affiliations:** 1 Department of Neurology, Washington University School of Medicine, St. Louis, MO USA; 2 Department of Pathology, Washington University School of Medicine, St. Louis, MO USA

**Keywords:** CNS cancer, Tumour immunology, CNS cancer

## Abstract

Brain tumors (gliomas) are heterogeneous cellular ecosystems, where non-neoplastic monocytic cells have emerged as key regulators of tumor maintenance and progression. However, relative to macrophages/microglia, comparatively less is known about the roles of neurons and T cells in glioma pathobiology. Herein, we leverage genetically engineered mouse models and human biospecimens to define the axis in which neurons, T cells, and microglia interact to govern Neurofibromatosis-1 (NF1) low-grade glioma (LGG) growth. *NF1*-mutant human and mouse brain neurons elaborate midkine to activate naïve CD8^+ ^T cells to produce Ccl4, which induces microglia to produce a key LGG growth factor (Ccl5) critical for LGG stem cell survival. Importantly, increased *CCL5* expression is associated with reduced survival in patients with LGG. The elucidation of the critical intercellular dependencies that constitute the LGG neuroimmune axis provides insights into the role of neurons and immune cells in controlling glioma growth, relevant to future therapeutic targeting.

## Abstract (web summary)

The role of neurons and T cells in glioma progression remains poorly understood. Here the authors show that midkine-dependent activation of a neuron-T cell-microglia axis promotes the growth of optic pathway gliomas.

## Introduction

Numerous studies in malignant solid tumors have highlighted the importance of non-neoplastic stromal cells, particularly cancer-associated fibroblasts, in tumor formation, growth, and progression^1,2^. However, in nervous system cancers, fibroblasts are not part of the tumor microenvironment, suggesting that other non-neoplastic cells, including neurons and immune cells (T cells, microglia, and macrophages), could serve this critical function. In this respect, both brain and nerve sheath tumors are characterized by the presence of neurons and infiltrating immune cells^3,4^, which could each provide cancer cell support.

These cellular interactions have been more extensively studied in malignant brain tumors (e.g. glioblastoma and diffuse midline glioma), where cancer cells attract resident microglia and peripheral macrophages through the elaboration of chemokines, which in turn produce growth factors that promote tumor growth and progression^5^. Similarly, neurons can also regulate malignant brain tumor growth involving several mechanisms, ranging from activity-dependent release of growth factors to tonic release of neurotrophins and neurotransmitters^6,7^.

In contrast, these stromal dependencies are likely to be greater in benign tumors with fewer genetic mutations, such as those arising in the setting of cancer predisposition syndromes, like neurofibromatosis type 1 (NF1), in which affected patients develop low-grade peripheral nerve sheath tumors and brain tumors (optic pathway gliomas; OPGs). Whereas peripheral nerve sheath tumors require growth factors from mast cells and macrophages^3,8^, NF1-OPGs depend on resident brain monocytes (microglia) for their formation and progression^9,10^. In *Nf1* murine optic gliomas, microglial production of a key growth factor (Ccl5) is both necessary and sufficient for tumor formation and growth^11,12^. Importantly, microglial Ccl5 expression requires T lymphocytes, such that glioma formation does not occur in mice lacking functional T cells^12^. However, it is currently not known how T cells are recruited to the developing tumor, how they are activated, and how their activation results in microglia Ccl5 production.

In light of the intimate association of these tumors with nerves and the increasing recognition that neurons can provide instructive signals to cancer cells, we sought to dissect the critical tumor-promoting axis involving neurons, immune cells, and low-grade gliomas (LGG) cancer cells using numerous converging cellular and molecular methodologies. Herein, we describe the complex cellular and molecular interactions between neurons, T cells, microglia, and glioma cells that comprise the LGG ecosystem, revealing critical roles for neurons and T cells in glioma formation and maintenance. We demonstrate that human and mouse *NF1*-mutant neurons produce midkine to activate T cells, which in turn leads to increased T cell Ccl4 secretion and the priming of microglia to elaborate Ccl5 to maintain LGG cell growth. Moreover, we identify each of the responsible intracellular signaling pathways and show that inhibiting integrin-mediated T cell brain entry or function is sufficient to attenuate glioma growth in vivo. The discovery of this co-dependent neuron/T cell/microglia/cancer cell axis provides opportunities for future pediatric LGG therapeutic targeting.

## Results

### T cell Ccl4 induces microglial Ccl5 production

**Fig. 1:**
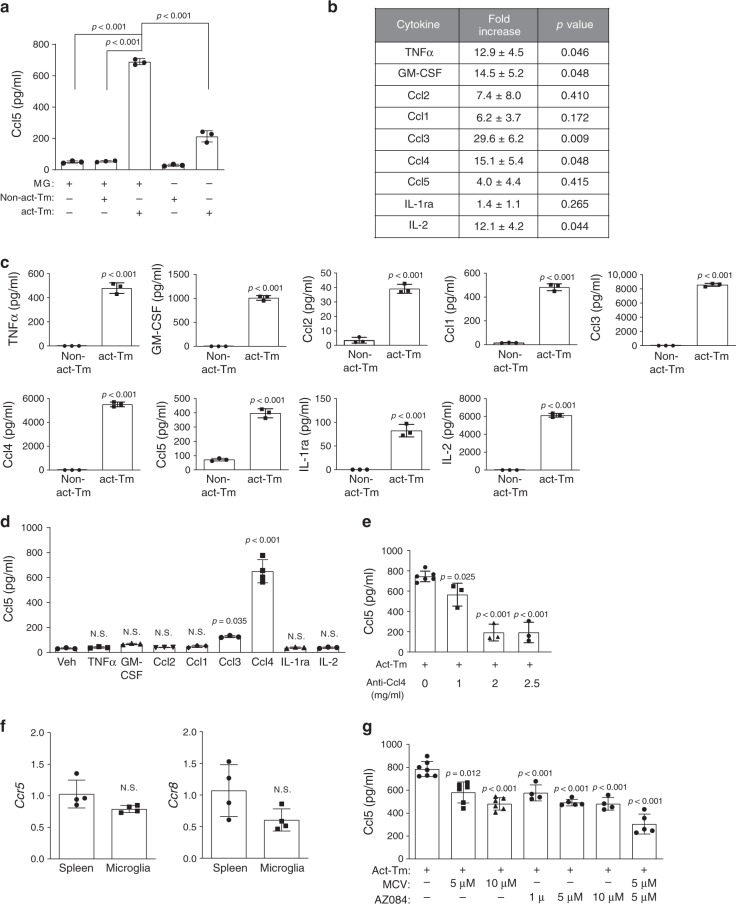
Activated T cells secrete Ccl4 and prime microglia to produce Ccl5. **a** Activated T cell conditioned medium (act-Tm CM) treatment stimulated WT microglia (MG) to produce a higher level of Ccl5 (ELISA) compared to non-activated T cell-conditioned medium (non-act-Tm CM). Non-activated and activated T cell CM, and CM collected from microglia alone served as controls for these experiments. **b** WT T cells isolated from the mouse spleen were seeded at the concentration of 2.5 × 10^6^ cells ml^−1^ in complete PRIM1640 medium followed by 2 days of CD3/CD28 stimulation (activated; act-Tm) or vehicle (PBS) treatment (non-activated; non-act-Tm). CM was collected for chemokine array triplicates. Increased levels of TNF-α, GM-CSF, Ccl2, Ccl1, Ccl3, Ccl4, Ccl5, Il-1ra, and Il-2 expression were observed in activated T cell CM relative to non-activated T cell CM. The fold increases and *P* values relative to control groups for all three replicates (Supplementary Fig. 1a) are collated in the table. **c** ELISA assays reveal increased levels of TNFα, GM-CSF, Ccl2, Ccl1, Ccl3, Ccl4, Ccl5, Il-1ra, and Il-2 in the CM of activated, relative to non-activated, T cells. **d** WT microglia were stimulated with these differentially expressed cytokines [TNF-α (400 pg ml^−1^), GM-CSF (1000 pg ml^−1^), Ccl2 (80 pg ml^−1^), Ccl1 (500 pg ml^−1^), Ccl3 (8000 pg ml^−1^), Ccl4 (6000 pg ml^−1^), Il-1ra (80 pg ml^−1^), and Il-2 (6000 pg ml^−1^)] for 24 h at the concentrations detected in the activated T cell CM. Ccl5 production by microglia was increased following Ccl4 (6000 pg ml^−1^) treatment. Veh: vehicle. **e** Ccl5 ELISA revealed that activated T cell CM induction of microglial Ccl5 production was reduced following treatment with increasing concentrations of Ccl4 neutralizing antibody. **f** Microglial *Ccr5* and *Ccr8* expression was validated using spleen as a positive control. **g** Increasing concentrations of maraviroc (MCV, Ccr5 receptor inhibitor) and AZ084 (Ccr8 receptor inhibitor) reduced T cell induction of microglial Ccl5 expression. The combination of MCV and AZ084 exhibited the greatest inhibition of microglial Ccl5 expression. All data are presented as the mean ± SEM. **a** This representative experiment was conducted with *n* = 3 independent biological samples, and was replicated two additional times with similar results. **b** *n* = 3 independent biological samples were examined over three independent experiments, as illustrated in Fig. S1a. **c** and **d** Bar graphs represent the means ± SEM of *n* = 3 independent biological samples. **e** This representative experiment was conducted with 0 mg ml^−1^ anti-Ccl_4_, *n* = 6; 1, 2, 2.5 mg ml^−1^ anti-Ccl4, *n* = 3, independent biological samples, and was replicated two additional times with similar results. **f** Bar graphs represent the means ± SEM of *n* = 4 independent biological samples. **g** This representative experiment was conducted with (from left to right) *n* = 7, *n* = 6, *n* = 6, *n* = 4, *n* = 5, *n* = 4, and *n* = 5 independent biological samples, and was replicated two additional times with similar results. **a**, **d**, **e**, **g** One-way ANOVA with Bonferroni post-test correction; **b**, **c**, **f** Two-tailed Student’s *t*-test. Exact *P* values are indicated within each panel; N.S.; not significant. From left to right in each panel: **a** all *P* < 0.001, **c** all *P* < 0.001, **d** N.S., N.S., N.S., N.S., *P* = 0.035, *P* < 0.001, N.S., N.S.; **e** *P* = 0.025, *P* < 0.001, *P* < 0.001; **f** N.S.; **g** *P* = 0.012, *P* < 0.001, *P* < 0.001, *P* < 0.001, *P* < 0.001, *P* < 0.001.

Previous studies from our laboratory demonstrated that only activated, but not naïve, T cells produce paracrine factors that induce microglia to produce Ccl5 (Fig. 1a)^10^. To identify the paracrine factors induced by T cell activation, we employed a commercially available cytokine array to query the conditioned medium (CM) from activated (anti-CD3/CD28-mediated activation) versus naïve (non-activated) T cells in triplicate. In total, nine cytokines/chemokines (i.e., TNFα, GM-CSF, Ccl2, Ccl1, Ccl3, Ccl4, Ccl5, IL-1ra, and IL-2) were increased in activated, relative to non-activated, T cell-CM (Fig. 1b and Supplementary Fig. 1a). We tested the ability of these cytokines to induce microglial Ccl5 production. After validating the increased expression of these nine cytokines, and the unaltered expression of two additional cytokines (*Il10* and *Ifnγ*) (Fig. 1c and Supplementary Fig. 1b) as negative controls, we treated WT microglia with each cytokine at the same concentrations measured in the activated T cell CM, and determined the microglia Ccl5 expression levels by ELISA. Only Ccl4 increased microglial Ccl5 protein expression to the levels seen following activated T cell CM treatment in both adult and neonatal microglia (Fig. 1a, d and Supplementary Fig. 1c, d). Similar results were obtained using different concentrations of these candidate cytokines (Supplementary Fig. 2a–h). While microglia were the major cell type expressing Ccl5 in the murine *Nf1* optic gliomas (Supplementary Fig. 1e), activated T cells produced some Ccl5 (Fig. 1a), which could contribute to the Ccl5 induction observed in our experimental paradigm. To exclude T cell Ccl5 from the observed microglial response, activated *Ccl5*^*−/−*^ T cells were analyzed. *Ccl5*-deficient T cell CM elicited a similar increase in microglial Ccl5 secretion, demonstrating that the Ccl5 produced by T cells did not contribute in this setting (Supplementary Fig. 2i).

Using the Immgen database, *Ccl4* expression is enriched in several T cell populations, including regulatory T cells (Tregs) and CD8^+^ T cells (Supplementary Fig. 2j). To determine whether Ccl4 is necessary for T cell CM-induced microglial Ccl5 production, a combination of Ccl4-neutralizing antibodies and Ccl4 receptor (Ccr5 and Ccr8) inhibitors were employed: Ccl4-neutralizing antibodies reduced activated T cell-induced microglia Ccl5 production by >60% (Fig. 1e). While both Ccr5 and Ccr8 were expressed by microglia (Fig. 1f), neither inhibiting Ccr5 (MCV treatment) or Ccr8 (AZ058 treatment) alone reduced Ccl5 to the same level as Ccl4-neutralizing antibodies (Fig. 1g). However, the combination of Ccr5 and Ccr8 inhibition (MCV + AZ058) reduced activated T cell-induced microglia Ccl5 production by ~60%, comparable to the effect observed with Ccl4-neutralizing antibodies (Fig. 1e, g). As controls, microglia were exposed to non-activated T cell CM in the presence or absence of Ccl4 receptor inhibition, with no effect on microglia Ccl5 production (Supplementary Fig. 2k). Since Ccl5 inhibits the apoptosis of *Nf1*-deficient high-grade glioma cells^13^, we analyzed the biological consequence of each of these reductions in Ccl5 expression on optic glioma stem cell (o-GSC) survival. Decreasing Ccl5 concentrations from 700 pg ml^−1^ to 400 or 200 pg ml^−1^, representing the Ccl5-inhibitory effect from anti-Ccl4 and Ccr5/Ccr8 blockers, increased o-GSC apoptosis by 50% or to untreated baseline levels, respectively (Supplementary Fig. 2l), thus establishing a clear biological effect of this Ccl4 axis inhibition on microglial support of LGG stem cell survival.

Ccl3 also increased microglia Ccl5 production, albeit to a far lesser extent (2.5-fold increase vs.18.5-fold increase in response to Ccl4; Fig. 1d). However, the combination of Ccl3 and Ccl4 together did not further increase microglial Ccl5 expression beyond what was seen with Ccl4 alone (Supplementary Fig. 2m). Taken together, these results establish Ccl4 as the cytokine responsible for inducing microglia Ccl5 production relevant to glioma growth regulation.

### MDK from *NF1*-mutant neurons induces T cell Ccl4 production

**Fig. 2:**
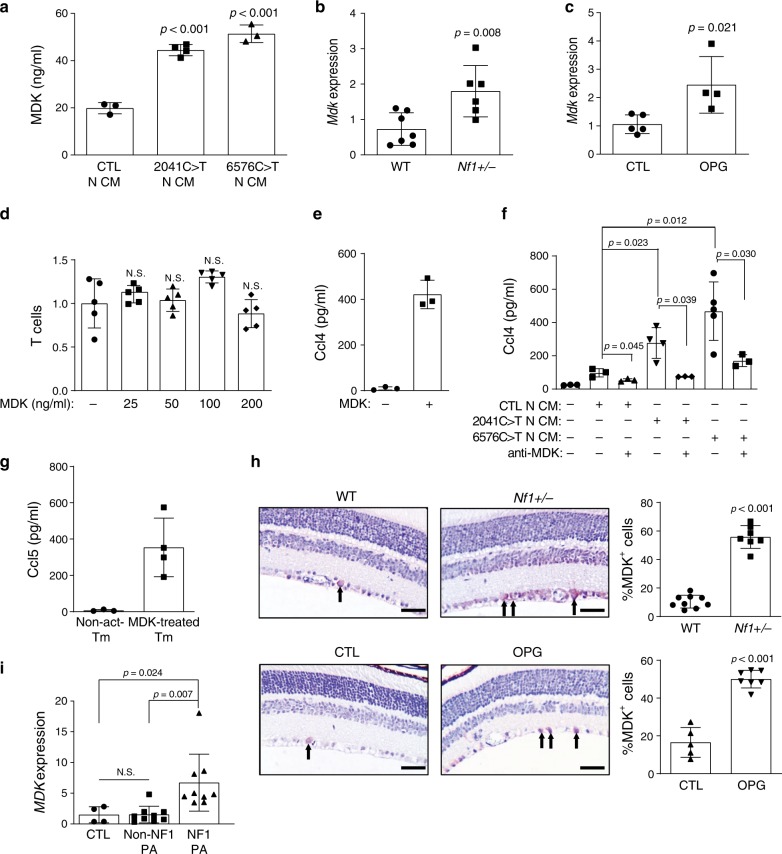
*NF1*-mutant neurons express MDK, which activates T cells to produce Ccl4. **a** Isogenic hiPSC-induced neurons with heterozygous NF1 patient *NF1* gene mutations (2041C>T and 6576C>T) produced higher levels of midkine in the neuron conditioned medium (N-CM) compared to WT (CTL) hiPSC-induced neurons. **b** *Mdk* gene expression was higher in the optic nerves of *Nf1*^+/−^ relative to WT mice. **c** Increased *Mdk* expression was observed in optic glioma (OPG)-containing relative to control (CTL) optic nerves. **d** No change in T cell migration was observed in response to various MDK concentrations. **e** MDK (50 ng ml^−1^) stimulation for 48 h increased T cell Ccl4 production. **f** CM from isogenic hiPSC**-**induced neurons with NF1 patient *NF1* gene mutations (c.2041C>T-N-CM and c.6576C>T-N-CM) exhibited a stronger T cell Ccl4 induction compared to CM from control hiPSC-induced neurons (CTL-N-CM). Anti-MDK neutralizing antibodies reduced T cell Ccl4 production in response to hiPSC-induced neuron CM stimulation. **g** MDK-activated (50 ng ml^−1^) T cell CM (mid-treated Tm) increased microglial Ccl5 production relative to non-activated T cell CM (non-act-Tm). **h** Immunohistochemistry revealed an increased percentage of MDK-immunoreactive cells in the retinal ganglion cell layer of OPG-bearing and *Nf1*^*+/−*^ mice relative to control (CTL) or WT mice, respectively. Scale bars, 40 µm. Arrows denote representative immunopositive cells. **i** *MDK* gene expression was examined in NF1 pilocytic astrocytomas (NF1-PAs, *n* = 9), non-NF1-PAs (*n* = 9), and non-neoplastic brain tissues (*n* = 4). Increased *MDK* expression was detected in NF1-PAs compared to normal brain tissue and non-NF1-PAs. Arrows denote representative immunopositive cells. All data are presented as the mean ± SEM. **a–c** These representative experiments were conducted with **a** CTL, *n* = 3, 2041C>T *n* = 4, 6576C>T, *n* = 3; **b** WT, *n* = 7, *Nf1*±, *n* = 6; **c** CTL, *n* = 5, OPG, *n* = 4, independent biological samples, and were replicated two additional times with similar results. **d** and **e** Bar graphs represent the means ± SEM of **d** *n* = 5, **e** *n* = 3, independ**e**nt biological samples. **f** This representative exp**e**riment was conducted with (from left to right) *n* = 3, *n* = 3, *n* = 3, *n* = 4, *n* = 3, *n* = 5, and *n* = 3 independent biological samples, and were replicated two additional times with similar results. **g** Bar graphs represent the means ± SEM of non-activated Tm, *n* = 3; MDK-treated Tm, *n* = 4 independent biological samples. **h** Bar graphs represent the means ± SEM of WT *n* = 9, *Nf1*^+/−^, *n* = 7, CTL, *n* = 5, OPG, *n* = 7, independent biological samples. **i** Bar graphs represent the means ± SEM of CTL, *n* = 4, non-NF1, PA, *n* = 9, NF1, PA, *n* = 9, independent biological samples. **a**, **d**, **f**, **i** One-way ANOVA with Bonferroni post-test correction, **b**, **c**, **h** Two-tailed Student’s *t*-test. Exact *P* values are indicated within each panel; N.S.; not significant. From left to right in each panel: **a** all *P* < 0.00, 1 **b** *P* = 0.008, **c** *P* = 0.021, **d** all N.S.; **f** top *P* = 0.012, middle *P* = 0.023, *P* = 0.030, bottom *P* = 0.045, *P* = 0.039; **i** CTL:NF1 PA *P* = 0.024, non-NF1 PA:NF1-PA *P* = 0.007; **h** all *P* < 0.001.

Since our experimental paradigm involved a non-physiologic method of T cell activation, we next sought to identify the factor(s) responsible for NF1-mediated T cell activation in the setting of *Nf1* OPG. For these studies, we leveraged both human-induced pluripotent stem cells (hiPSCs) harboring actual NF1 patient germline *NF1* gene mutations, as well as *Nf1*-mutant mice. Human neurons were generated from isogenic hiPSCs either heterozygous (*NF1*-het) or homozygous (*NF1*-null) for two NF1 patient germline *NF1* gene mutations [c.2041C>T and c.6576C>T] using established protocols^13^. In light of previous work demonstrating elevated midkine (MDK) levels in NF1 patient samples, including low-grade peripheral nerve sheath tumors (neurofibromas^14^) and skin^15^, we employed a commercial array containing MDK and other cytokines. Using this assay, *NF1*-null neurons secreted higher levels of MDK and CSF-2 relative to control neurons (Supplementary Fig. 3a). Quantitative real-time polymerase chain reaction (qRT-PCR) validation revealed increased *MDK*, but not *CSF2*, mRNA expression in *NF1*-null, relative to control, neurons (Supplementary Fig. 3b, c). In contrast, increased CSF-2 protein levels were detected by ELISA in the CM of both *NF1*-null neurons relative to control neurons, but at much lower levels relative to MDK [CSF-2: 100–300 pg ml^−1^ (Supplementary Fig. 3d), MDK: 40,000–50,000 pg ml^−1^ (Fig. 2a)].

Neuron-derived MDK or CSF-2 could influence T cell biology in two ways. First, it could function as a chemoattractant for T cells. However, CD3^+^ T cell migration measured using a transwell assay was not increased in response either to MDK or CSF-2 (Fig. 2d**;** Supplementary Fig. 3e). Second, it is possible that MDK or CSF-2 activates T cells to produce Ccl4. For these experiments, we stimulated CD3^+^ T cells with either MDK or CSF-2 at the concentrations measured in the CM of heterozygous *NF1*-mutant hiPSC-derived neurons (~50 ng ml^−1^ MDK, 200 pg ml^−1^, CSF-2), as seen in patients with NF1. While T cells exhibited increased Ccl4 production in response to MDK stimulation (Fig. 2e), there was no Ccl4 induction following CSF-2 treatment (Supplementary Fig. 3f), thus excluding CSF-2 as a potent neuronal T cell activator.

To determine whether MDK mediates hiPSC-derived neuron CM to increase T cell Ccl4 release, T cells were incubated with CM collected from control and heterozygous *NF1*-mutant (c.2041C>T and c.6576C>T) hiPSC-derived neurons. Treatment with the CM of c.2041C>T and c.6576C>T *NF1*-mutant hiPSC-neurons increased T cell Ccl4 production (Fig. 2f). MDK mediated this induction, since MDK-neutralizing antibodies attenuated the neuron CM-induced Ccl4 increase (Fig. 2f). It should be noted that *NF1*-mutant neuron CM contains a similar concentration of MDK (50 ng ml^−1^) to the commercially purified MDK used for T cell treatment (Fig. 2e). The fact that hiPSC *NF1-*mutant neuron CM was diluted in T cell CM (30% v/v) in order to maintain healthy T cell cultures, reduced the overall concentration of MDK by three-fold compared to the purified MDK treatments, thus resulting in relatively attenuated Ccl4 induction. Consistent with MDK as the primary activation signal for T cell priming of microglia, CM from MDK-treated T cells induced microglial Ccl5 production to an equivalent extent after normalizing to cell number and medium volume (Fig. 2g). Finally, we sought to define the mechanism underlying *NF1* protein (neurofibromin) control of neuronal MDK expression. As the main function of the *NF1* protein is to negatively regulate RAS activity, we treated control and *NF1*-mutant hiPSC-induced neurons with lovastatin (a crude RAS inhibitor) at different concentrations^16^. Following lovastatin treatment, MDK expression in these *NF1*-mutant hiPSC-induced neurons was reduced (Supplementary Fig. 3g), without alterations in neuronal survival (Supplementary Fig. 3h).

Relevant to human and mouse NF1-LGG, we also examined MDK expression in the *Nf1* OPG mice and human NF1-LGG (pilocytic astrocytoma; PA) samples. Consistent with the hiPSC* NF1-*mutant neuron findings (Fig. 2a), optic nerves from *Nf1*^+/−^ and *Nf1* OPG mice exhibited higher levels of *Mdk* transcript expression (Fig. 2b, c), and a greater percentage of MDK-expressing cells in the retinal ganglion cell layer of the retina relative to control mice (Fig. 2h). In addition, *MDK* transcript expression was elevated in NF1-PA samples relative to non-NF1-PA and normal brain tissue (Fig. 2i). Taken together, these results establish increased MDK expression as a common feature unique to mouse and human NF1-LGGs, not shared with non-NF1-PAs, which functions as an activator of T cells to produce Ccl4.

### MDK induces T cell Ccl4 via Lrp1/calcineurin/NFAT1 signaling

**Fig. 3:**
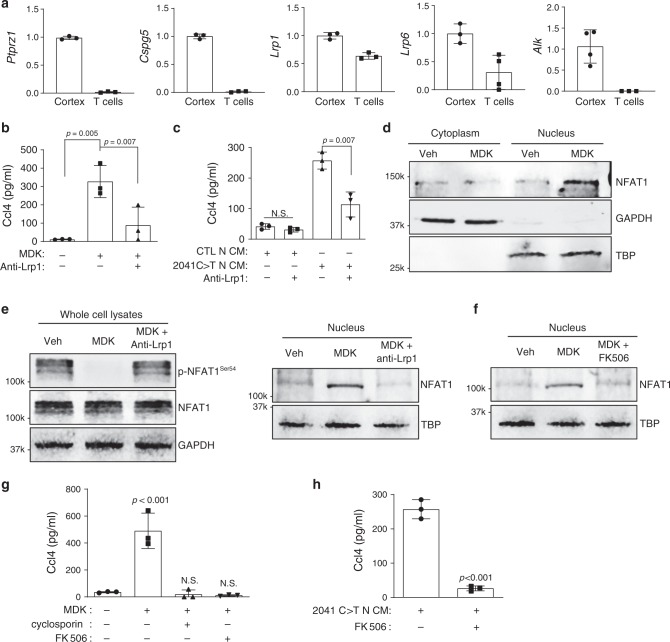
MDK activates T cells to produce Ccl4 through Lrp1/calcineurin/NFAT1 signaling. **a** T cells expressed only two (Lrp1 and Lrp6) of the putative MDK receptors [protein-tyrosine phosphatase ζ (*Ptprz1*), neuroglycan-C (*Cspg5*), low density-lipoprotein receptor-related protein-1 (*Lrp1*), low density-lipoprotein receptor-related protein-6 (*Lrp6*) and anaplastic lymphoma kinase (*Alk*)] by quantitative RT-PCR. Normal mouse cortex was used as an internal positive control. **b** Lrp1 blocking antibodies (30 µg ml^−1^) reduced MDK-induced Ccl4 production in T cells. **c** The 2041C>T neuron conditioned media (N-CM)-mediated Ccl4 production in T cells was attenuated following exposure to Lrp1 receptor blocking antibodies. **d** Immunoblotting revealed increased NFAT1 nuclear  localization in T cells following MDK treatment. Glyceraldehyde 3-phosphate dehydrogenase (GAPDH) and TATA-binding protein (TBP) served as loading controls for the cytoplasm and nuclear fractions, respectively. **e** Decreased levels of phosphorylated-NFAT1 (p-NFAT) were observed after MDK stimulation of T cells. Lrp1 blocking antibodies (anti-Lrp1, 30 µg ml^−1^) increased NFAT1 phosphorylation and impaired NFAT1 nuclear localization in MDK-stimulated T cells. **f** The calcineurin inhibitor FK506 (10 μM) inhibited MDK-induced NFAT1 nuclear localization. **g** Calcineurin inhibitors, cyclosporin (100 nM) and FK506 (10 μM), inhibited MDK-induced Ccl4 production in T cells. **h** FK506 (10 μM) reduced 2041C>T neuron conditioned media (N-CM)-induced Ccl4 production in T cells. All data are presented as the mean ± SEM. **a** Bar graphs represent the means ± SEM of *n* = 3 independent biological samples. **b**, **c**; **g**, **h** These representative experiments were conducted with *n* = 3 independent biological samples,and were replicated two additional times with similar results. **b**, **c**, **g** One-way ANOVA with Bonferroni post-test correction, **h** Two-tailed Student’s *t*-test. Exact *P* values are indicated within each panel; N.S.; not significant. From left to right in each panel: **b** *P* = 0.005, *P* = 0.007; **c** N.S., *P* = 0.007; **g** *P* < 0.001, N.S., N.S.; **h** *P* < 0.001. **d**–**f** These are representative images of *n* = 3 independent biological samples examined over three independent experiments with similar results. Molecular weight markers are denoted at the left side of each blot.

To determine how MDK activates T cells, we examined the expression of its known receptors, including Ptprz1^17^, Cspg5^18^, Lrp1, Lrp6^19^, and Alk^20^. By qRT-PCR, T cells express abundant *Lrp1* (Fig. 3a). To determine whether Lrp1 was necessary for MDK-mediated Ccl4 secretion from T cells, we incubated T cells with MDK or hiPSC* NF1-*mutant neuron CM in the presence of Lrp1-neutralizing antibodies. Lrp1-neutralizing antibodies inhibited the ability of both MDK and hiPSC* NF1-*mutant neuron CM to increase T cell Ccl4 expression (Fig. 3b,c). MDK has been reported to signal through multiple different intracellular pathways involving Akt, Atf^21^, Stat4, NFAT1, NFAT2^22^, and Src^23^. While no differences in Akt, Atf, Stat4, NFAT2 activation were observed (Supplementary Fig. 4), increased nuclear NFAT1 levels were detected following MDK exposure (Fig. 3d). This nuclear translocation results from dephosphorylation of the serine-rich region (Serine-54) within the amino termini of NFAT1 proteins, and the following conformational change that exposes a nuclear localization signal^24^. As such, MDK treatment also dephosphorylated NFAT1 (Fig. 3e). Importantly, Lrp1 inhibition increased phosphorylated NFAT1^S54^ levels and blocked the nuclear translocation of NFAT1 in the setting of MDK exposure (Fig. 3e). Lrp1 can activate NFAT1 through ERK and calcineurin^25^. While we observed no change in ERK activity after MDK exposure (Supplementary Fig. 4), NFAT1 nuclear transport was reduced following calcineurin inhibition^26^ (FK506; Fig. 3f). Moreover, MDK-induced T cell Ccl4 production was reduced following treatment with the FK506 and cyclosporin (calcineurin inhibitors^24^; Fig. 3g). FK506 treatment also inhibited hiPSC-derived neuron CM induction of T cell Ccl4 production (Fig. 3h). Collectively, our findings demonstrate that MDK binds to Lrp1 on T cells and induces Ccl4 expression through calcineurin-mediated NFAT1 nuclear translocation.

### CD8^+^ T cell brain entry is required for optic glioma growth

**Fig. 4:**
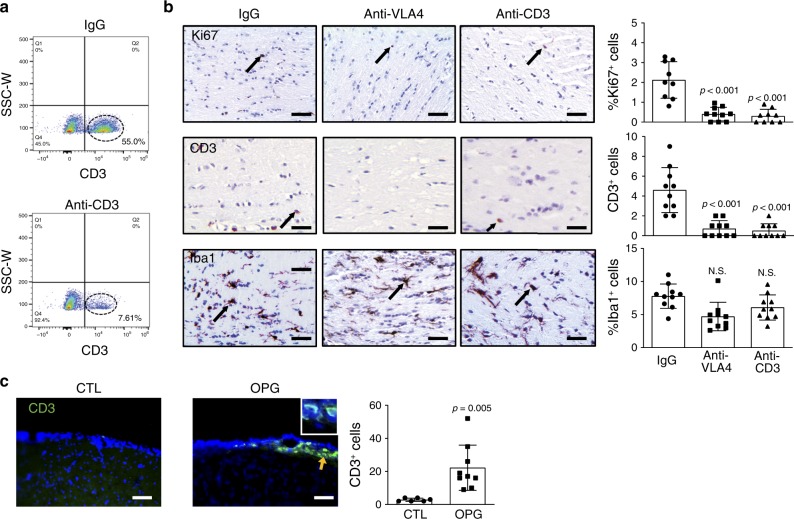
*Nf1* optic glioma mice exhibit increased meningeal and parenchymal T cells. **a** CD3^+^ T cells (circled with dash line) were depleted in splenocytes from anti-CD3 antibody-treated mice (7.6%) relative to IgG-treated mice (55%), as measured by flow cytometry. **b** Immunohistochemistry revealed that anti-VLA4 and anti-CD3, but not control IgG, antibody treatments reduced CD3^+^ T cell infiltration and the percentage of Ki67^+^ cells in murine *Nf1* optic gliomas. No differences in microglia content (%Iba1^+^ cells) were observed in the anti-VLA4 and anti-CD3 groups compared to the IgG controls. Arrows denote representative immunopositive cells. Black arrows indicate representative immunopositive cells. **c** Immunofluorescence microscopy revealed increased meningeal CD3^+^ lymphocyte infiltration in optic glioma (OPG)-bearing mice, relative to the control (CTL) mice. Yellow arrow denotes representative immunopositive cells. DAPI (blue) is used as a nuclear counter stain. **a**, **c** Scale bars, 40 µm. **b** Bar graphs represent the means ± SEM of (top panel) IgG, *n* = 9; anti-VLA4, *n* = 10; anti-CD3, *n* = 9; (middle and bottom panels), all groups had *n* = 10 independent biological samples. **c** Bar graphs represent the means ± SEM of CTL, *n* = 6; OPG, *n* = 9; independent biological samples. **b** One-way ANOVA with Bonferroni post-test correction; **c** Two-tailed Student’s *t*-test. Exact *P* values are indicated within each panel; N.S.; not significant. From left to right in each panel: **b** top panel: all *P* < 0.001, middle panel: all *P* < 0.001, bottom panel: all N.S.; **c** *P* = 0.005.

While the above studies revealed important roles for neurons and T cells in LGG stroma-tumor circuitry, they do not establish whether T cells are necessary for optic glioma growth. To demonstrate a requirement for T cells and directed T cell infiltration in murine *Nf1* optic glioma growth, we performed two preclinical proof-of-principle experiments using neutralizing antibodies. First, *Nf1* OPG mice were treated with anti-CD3 antibodies to deplete T cells from 6 weeks of age until 3 months of age, verified by flow cytometry of splenic cells (IgG group, 55% CD3^+^ cells; anti-CD3 group, 7.61%; Fig. 4a). Relative to isotype antibody (IgG) treatments, the OPGs in these mice had reduced numbers of CD3^+^ T cells and attenuated tumor proliferation (Fig. 4b). Second, since T cells are rarely found in the normal brain parenchyma, but are detected in NF1-associated mouse and human LGGs^12,27^, we examined the meningeal space in control (CTL, *Nf1*^*flox/flox*^) and *Nf1* optic glioma (OPG, *Nf1*^*flox/mut*^; GFAP-Cre) mice as a potential point of entry^28^. Similar to the optic nerves^27^, more CD3^+^ T cells were observed in the meningeal space of OPG mice relative to controls (Fig. 4c), suggesting that T cells in the setting of optic gliomas are most likely recruited from this location into the evolving tumor. Because T cell migration from the meningeal space is mediated by integrins, such as the very late antigen 4 (VLA4)^29^, *Nf1* OPG mice were treated with anti-VLA4 antibodies in a parallel experiment. Consistent with a dependency on integrin for T cell parenchymal invasion, anti-VLA4 treatment inhibited CD3^+^ T cell tumor infiltration, and reduced tumor proliferation (Fig. 4b). Interestingly, neither treatment affected microglia content (Iba1^+^ cells, Fig. 4b).

**Fig. 5:**
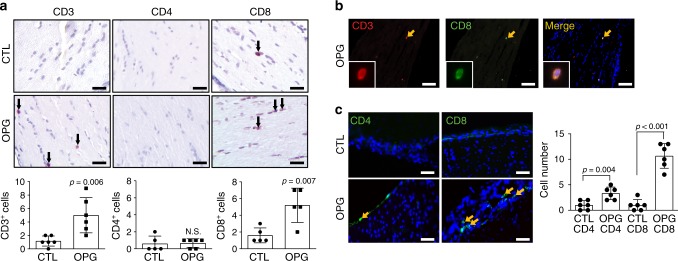
*Nf1* optic glioma mice exhibit increased numbers of CD8^+^ T cells in the meninges and optic nerve. **a** Increased CD3^+^ and CD8^+^ T cell infiltration was observed in *Nf1* optic glioma (OPG)-containing nerves relative to control (CTL) optic nerves. Few CD4^+^ T cells were detected in murine *Nf1* OPG or control optic nerves. Black arrows denote representative immunopositive cells. Scale bars, 20 µm. **b** CD3^+^ (red); CD8^+^ (green) double-positive cells were found (yellow arrow) in murine *Nf1* optic gliomas. **c** Increased numbers of meningeal CD4^+^ and CD8^+^ T cells (green) were present in *Nf1* OPG mice relative to controls, where the number of CD8^+^ T cells was three-fold higher than CD4^+^ T cells. DAPI (blue) is used as a nuclear counter stain. Yellow arrows denote representative immunopositive cells. **b**, **c** Scale bars, 40 µm. **a**, **c** Bar graphs represent the means ± SEM of **a** (left panel) *n* = 6, (middle panel) CTL, *n* = 5; OPG, *n* = 6, (right panel) *n* = 5 or **c** *n* = 6 independent biological samples. **a** Two-tailed Student’s *t*-test, **c** One-way ANOVA with Bonferroni post-test correction. Exact *P* values are indicated within each panel; N.S.; not significant. From left to right in each panel: **a** left panel *P* = 0.006, middle panel N.S., right panel *P* = 0.007; **c** *P* = 0.004, *P* < 0.001.

Next, we sought to determine which T cell population predominated in these tumors using CD4, CD8, and Foxp3 antibodies. Whereas no Foxp3^+^ cells were detected in the OPGs (Supplementary Fig. 5a), the majority of the T cells in the tumors were CD8^+^ cells, rather than CD4^+^ cells (Fig. 5a). The cellular identity of these CD8^+^ cells in the mouse *Nf1* OPGs were verified as CD3^+^ T cells by double labeling immunohistochemistry (Fig. 5b). A similar pattern was also observed in the meninges of these mice, where the vast majority of the T cells were CD8^+^, with a smaller infiltration of CD4^+^ cells (Fig. 5c). Consistent with these findings in murine OPGs, CD8^+^ T cells were also detected in human NF1-PAs (*N* = 4; Supplementary Fig. 5b).

**Fig. 6:**
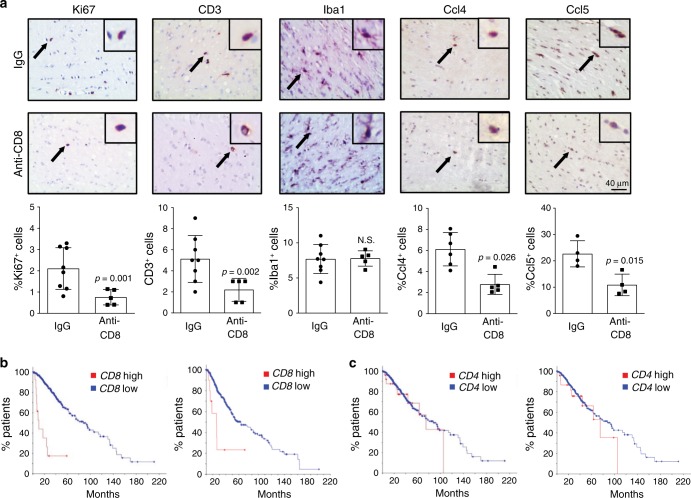
CD8^+^ T cells control *Nf1* optic glioma growth. **a** Immunohistochemistry revealed that anti-CD8 antibody treatment reduced the number of CD3^+^ T cells, as well as the percentage of Ki67^+^ cells, Ccl4^+^ cells, and Ccl5^+^ cells, in *Nf1* optic glioma specimens. Black arrows denote representative immunopositive cells. Scale bar, 40 µm. No differences in microglia (%Iba1^+^ cells) content were observed in anti-CD8 treated mice compared to the IgG control group. Bar graphs represent the means ± SEM of %Ki67^+^ cells, IgG, *n* = 8; anti-CD8, *n* = 5; CD3^+^ cells, IgG, *n* = 8; anti-CD8, *n* = 5; %Iba1^+^ cells, IgG, *n* = 8; anti-CD8, *n* = 5; %Ccl4^+^ cells, IgG, *n* = 6; anti-CD8, *n* = 5; %Ccl5^+^ cells, IgG, *n* = 4; anti-CD8, *n* = 4, independent biological samples. Two-tailed Student’s *t*-test. Exact *P* values are indicated within each panel; N.S.; not significant. From left to right in each panel: **a** % Ki67^+^ cells, *P* = 0.001; CD3^+^ cells, *P* = 0.002; %Iba1^+^ cells, N.S.; %Ccl4^+^ cells, *P* = 0.026; % Ccl5^+^ cells, *P* = 0.015. **b** Kaplan–Meier survival analysis (Brain Lower Grade Glioma TCGA Provisional [left panel; *P* = 6.72e–13] and TCGA PanCancer Atlas [right panel; *P* = 7.11e−14] datasets) demonstrates that non-overlapping patients with LGG and high tumor *CD8* expression have shorter survival time, while **c** high *CD4* expression was not associated with reduced survival time (Brain Lower Grade Glioma TCGA Provisional [left panel; *P* = 0.371] and TCGA PanCancer Atlas [right panel; *P* = 0.598] datasets).

To establish a requirement for CD8^+^ cells in *Nf1* OPG growth, we employed anti-CD8 antibody treatment to deplete CD8^+^ T cells using a protocol similar to that described for the anti-CD3 treatments. CD8^+^ T cell depletion was validated in splenocytes by FACS analysis (IgG group, 50.3% CD8^+^ cells; anti-CD3 group, 0.18%; Supplementary Fig. 5c). As observed following CD3 antibody exposure, the OPGs in treated mice had reduced numbers of CD3^+^ T cells and attenuated tumor proliferation, but no change in microglia content (Fig. 6a). Consistent with a key role for this population of T lymphocytes in LGG pathobiology and similar to CD3^+^ T cells, CD8^+^ T cells produced Ccl4 in response to MDK (Supplementary Fig. 5d), which was inhibited by MDK-neutralizing antibodies (Supplementary Fig. 5d). Additionally, microglia exposed to MDK-treated CD8^+^ T cell CM exhibited similar levels of microglial Ccl5 (Supplementary Fig. 5e) as observed following MDK-treated CD3^+^ T cell CM exposure (Fig. 2g). Consistent with the T cell Ccl4 regulation of microglial Ccl5 expression, anti-CD8 antibody treatment of *Nf1* OPG mice reduced the percent of Ccl4^+^and Ccl5^+^cells in the murine LGGs (Fig. 6a).

Lastly, to explore the possible clinical relevance of CD8^+^ T cell infiltration, we analyzed *CD8* and *CD4* RNA expression in datasets with LGG [Brain Lower Grade Glioma (TCGA, Provisional, overlapping patients excluded) and Brain Lower Grade Glioma (TCGA, PanCancer Atlas) samples]^30^. In both LGG datasets, shorter overall survival correlated with increased *CD8*, but not *CD4*, expression (Fig. 6b, c). Similarly, increased *CD8* expression also correlated with reduced progression-free survival in the one dataset (TCGA, Provisional) with sufficient numbers of samples for analysis (Supplementary Fig. 5f). Taken together, these findings demonstrate that CD8^+^ T cell entry into the brain is required for *Nf1 *OPG growth, which is also associated with poor clinical outcome in patients with LGG.

### T cell-induced microglial Ccl5 production inhibits apoptosis

Additionally, we sought to elucidate the mechanisms responsible for microglia Ccl5 production and LGG cancer cell growth. Initial experiments focused on defining the monocyte population (macrophages versus microglia) and the effect of a germline *Nf1* gene mutation on the involved monocyte population. First, while the monocytes that populate the murine *Nf1* OPGs are CD11b^+^/CD45^low^/Cx3cr1^+^ cells (microglia)^9^, rather than peripheral CD11b^+^/CD45^high^/Cx3cr1^-^ macrophages, we nevertheless examined the responses of WT splenic monocytes (macrophages) to activated T cell CM (Supplementary Fig. 6a). In striking contrast to the results obtained with microglia, no significant induction of Ccl5 in splenic monocytes was observed, supporting a T cell-microglia, rather than T cell-macrophage, circuit in the maintenance of *Nf1* OPGs. Second, because the stromal (T cells and microglia) cells in both murine and human NF1-OPG are heterozygous for a germline *NF1* gene mutation, we also examined the ability of *Nf1*^+*/*−^ T cells and microglia to interact and produce Ccl5. Similar induction of microglial Ccl5 was observed using activated *Nf1*^+/−^ T cell CM or *Nf1*^+*/*−^ microglia (Supplementary Fig. 6b, c). For these reasons, we explored the regulation of Ccl5 using WT T cells and microglia.

**Fig. 7:**
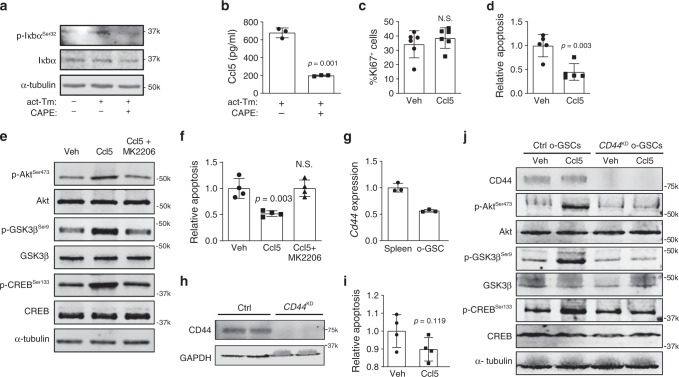
T cell-mediated microglial Ccl5 production inhibits LGG apoptosis through CD44. **a** Activated T cell CM (act-Tm) increased Iκbα phosphorylation in microglia, which was reduced following Caffeic acid phenethyl ester (CAPE; 100 µM) treatment. **b** CAPE treatment attenuated activated T cell CM (act-Tm)-mediated Ccl5 production in microglia. OPG o-GSCs were stimulated with Ccl5 at 300 pg ml^−1^ for 3 days. Whereas no increased optic glioma stem cell (o-GSC) proliferation (%Ki67^+^ cells) was observed **c**, o-GSC apoptosis (%TUNEL^+^ cells) was reduced in the Ccl5-treated group compared to vehicle (Veh, 0.1% BSA) treated controls **d**. **e** Immunoblotting reveals activation of the Akt-GSK3β-CREB anti-apoptosis pathway following Ccl5 treatment, which was inhibited by AKT inhibitor (20 μM MK2206) treatment. **f** 20 μM MK2206 treatment inhibited the anti-apoptosis effect of Ccl5 on o-GSCs (%TUNEL^+^ cells). **g** *Cd44* expression in o-GSCs was detected by qRT-PCR using splenocytes as a positive control. *Cd44* expression differences were not statistically analyzed. **h** *CD44* knockdown (*CD44*^KD^) in o-GSCs reduces CD44 expression relative to the control shRNA-treated (Ctrl) cells. **i** Apoptosis (%TUNEL^+^ cells) of *CD44*^KD ^o-GSCs was not inhibited by Ccl5 treatment. **j** Immunoblotting reveals that Ccl5 activation of the Akt/GSK3β/CREB anti-apoptosis pathway was inhibited by *CD44* knockdown. All data are presented as the mean ± SEM. **b**–**d**, **f**, **i** These representative experiments were conducted with **b** *n* = 3, **c** *n* = 6, **d** *n* = 5; **f**, **i** *n* = 4 independent biological samples and were replicated two additional times with similar results. **g** Bar graphs represent the means ± SEM of *n* = 3 independent biological samples. **b**–**d**, **i** Two-tailed Student’s *t*-test; **f** One-way ANOVA with Bonferroni post-test correction. Exact *P* values are indicated within each panel; N.S.; not significant. From left to right in each panel: **b** *P* = 0.001; **c** N.S.; **d** *P* = 0.003; **f** *P* = 0.003, N.S.; **i** *P* = 0.119. **a**, **e**, **h**, **j** These are representative images of *n* = 3 independent biological samples examined over three independent experiments with similar results. Molecular weight markers are denoted at the right side of each blot.

Since NFκB has been previously implicated in *Ccl5* transcriptional regulation^31^, we examined NFkB inhibitor (Iκbα) phosphorylation in microglia following exposure to activated T cell CM. Increased Ikbα phosphorylation, indicating higher levels of NFκb activity, was observed following activated T cell CM treatment, which was abrogated using an NFκB inhibitor (Caffeic acid phenethyl ester, CAPE) (Fig. 7a). Importantly, increased microglial Ccl5 production in response to activated T cell CM exposure was blocked by NFκB inhibition (CAPE; Fig. 7b).

Based on prior studies demonstrating that Ccl5 is necessary for murine *Nf1* OPG growth in vivo^11,12^ and operates to control glioblastoma cell growth by inhibiting apoptosis, we next sought to determine whether Ccl5 increases the survival of cancer stem cells from murine *Nf1* optic gliomas (optic glioma stem cells; o-GSCs). We focused on these cells, since they are capable of generating glioma-like lesions in mice following transplantation, and no suitable primary human NF1-LGG cell lines are currently available for analysis. Whereas Ccl5 treatment in the growth phase (non-confluent) did not change the percentage of proliferating (Ki67^+^) o-GSCs (Fig. 7c), Ccl5 reduced the apoptosis of confluent o-GSC cultures (TUNEL staining; Fig. 7d).

To identify the signaling pathway underlying Ccl5 control of o-GSC apoptosis, we performed a survey of known *NF1* protein (neurofibromin) downstream effectors. While no change in ERK activity was observed following Ccl5 treatment (Supplementary Fig. 6d), Akt and CREB phosphorylation was increased (Fig. 7e). Since Akt activates the CREB pathway through GSK3β in hepatocytes^32^, we measured GSK3β activity. Consistent with Akt/GSK3β/CREB hyperactivation as the responsible pathway underlying Ccl5-mediated o-GSC survival, Akt inhibition (MK2206) following Ccl5 treatment attenuated Akt, GSK3β, and CREB activation (using phospho-specific antibodies), as well as increased o-GSC apoptosis (TUNEL) (Fig. 7e, f).

We have previously shown that CD44 is the CCL5 receptor on *Nf1*-deficient high-grade glioma cells^33^. To determine whether Ccl5 functions through this receptor, we first analyzed o-GSC CD44 mRNA and protein expression (Fig. 7g,h). Second, following *Cd44* knockdown in o-GSCs, Ccl5 no longer inhibited o-GSC death (Fig. 7h–j). As such, Ccl5 operates to increase low-grade and high-grade glioma survival through CD44 binding and downstream signaling.

**Fig. 8:**
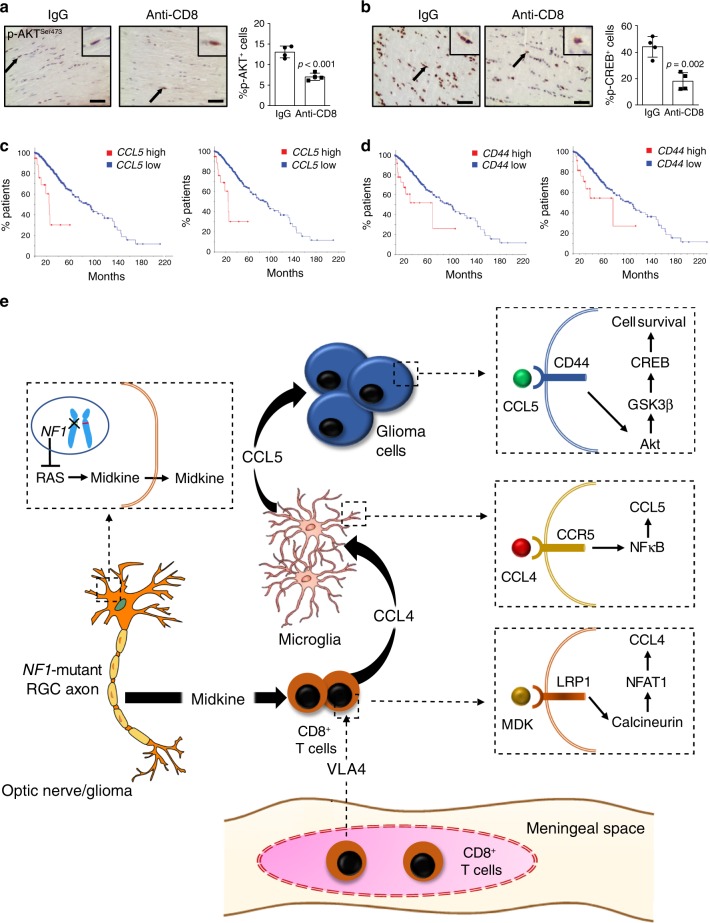
T cell-induced Ccl5/CD44-mediated cell survival underlies *Nf1* optic glioma growth. **a**, **b** Immunohistochemistry revealed that anti-CD8 antibody treatment reduced the percentage of p-AKT^Ser473^-expressing and p-CREB^Ser133^-expressing cells in mouse *Nf1* optic glioma specimens. Black arrows denote representative immunopositive cells. Scale bars, 20 µm. Bar graphs represent the means ± SEM of *n* = 4 independent biological samples. Two-tailed Students-*t* test. Exact *P* values are indicated within each panel; **a** *P* < 0.001; **b** *P* = 0.002. **c**, **d** Kaplan–Meier survival curves (Brain Lower Grade Glioma TCGA Provisional [2 left panels; **c** *P* = 1.29e−6, **d** *P* = 2.06e−3] and TCGA PanCancer Atlas [2 right panels; **c** *P* = 4.69e−13, **d** *P* = 9.86e−3] datasets) demonstrate that non-overlapping patients with LGG who harbor high *CCL5* expression or *CD44* expression have reduced survival time. **e** Schematic representation of the neuron–immune–cancer axis in NF1-LGG. Meningeal T cells infiltrate into the optic glioma in an integrin (VLA-4)-dependent manner, and are activated by MDK produced by *Nf1*-mutant retinal ganglion cells (neurons) through a RAS-dependent mechanism. This neuron-mediated T cell activation increases CD8^+^ T cell Ccl4 production through increased Lrp1/calcineurin signaling, and results in increased NFκB-dependent microglial Ccl5 expression, culminating in increased glioma growth through Akt/GSK3β/CREB pathway-mediated suppression of cancer (glioma) stem cell apoptosis and increased tumor growth.

Since Ccl5 mediates o-GSC survival via the AKT/GSK3β/CREB pathway, we examined the percentage of p-AKT^Ser473^-immunopositive cells and p-CREB^Ser133^-immunopositive cells following anti-CD8 treatment in *Nf1* optic glioma mice. Consistent with T cell Ccl4 induction of microglial Ccl5-mediated optic glioma growth, we observed a reduction in the percentage of p-AKT^Ser473^-immunopositive cells and p-CREB^Ser133^-immunopositive cells in the optic gliomas of anti-CD8 antibody-treated mice (Fig. 8a, b).

Finally, in keeping with a key role for Ccl5 and CD44 in LGG, high *CCL5* or *CD44* expression also correlated with reduced overall survival in the two available TCGA datasets with LGGs (overlapping patients excluded; Fig. 8c, d). A similar correlation between high tumor *CCL5* expression and reduced progression-free survival was observed in the one dataset with sufficient numbers of samples for analysis; Supplementary Fig. 6e).

## Discussion

Using NF1 as a genetic model system to study low-grade brain tumor pathobiology, we found that the glioma microenvironment is composed of immune (T cells and microglia) and non-immune (neurons) stromal cells, which together establish fertile soil supportive of the development and progression of these tumors. In this regard, we show that CD8^+^ T cells are required for glioma growth, such that inhibiting their entry into the brain attenuates tumor proliferation (VLA4, CD3, and CD8 antibody treatments). Moreover, we demonstrate that *NF1*-mutant neurons support glioma growth by producing MDK, which activates T cells to produce Ccl4, a cytokine that induces microglia to express a critical glioma growth factor (Ccl5) (Fig. 8e). These findings raise several important points relevant to solid tumor pathobiology.

While traditionally viewed as bystander cells in the pathogenesis of cancer, recent studies argue that neurons provide instructive cues for tumor formation and growth. First, neurons secrete neurotransmitters and neuropeptides, which can bind to their cognate receptors on tumor cells to directly increase brain tumor cell growth^34^. Second, using optogenetic approaches, increased neuronal activity has been shown to drive high-grade glioma growth through the shedding of neuroligin-3, a protein normally involved in neuron–neuron communication^6^. Third, nerve innervation is a poor prognostic marker in many cancers outside of the nervous system (pancreas and prostate cancer)^35,36^ such that neuron-secreted factors promote tumor initiation, growth, and metastasis^37,38^. For example, autonomic nerve fibers positively mediate prostate cancer initiation and progression through adrenergic and cholinergic signaling pathways^37^. In addition, neural progenitor cells (NPCs) can migrate from the brain to prostate tumors, and drive tumor proliferation and metastasis^39^.

In addition to directly mediate tumor growth, neurons can also contribute to tumorigenesis by modulating immune cell function. In the brain, neurons produce membrane-bound and soluble factors (e.g., CD22^40^, CD200^41^, ICAM-5^42^, semaphorin-3A^43^, and CX3CL1^44^) that each can modulate microglia properties. These tightly regulated interactions are not only essential for normal brain development, during which neuronal complement (C1q) expression triggers microglia-mediated synaptic pruning, but can be subverted to drive nervous system disease progression in the setting of peripheral nerve injury^45^ and neurodegenerative disorders^46^. Similarly, neurons produce TGFβ to increase CD4^+^ T cell proliferation^47^, as well as Fas ligand to promote T cell apoptosis^48^.

Our finding that *NF1*-mutant mouse and human neurons increase their expression of MDK, which operates to activate T cells through NFAT-1 signaling, also expands the role of neurons as immune system regulators. In this regard, neuronal shedding of intercellular adhesion molecule (ICAM)-5 inhibits the activation of naïve T cells^49^. While further studies will be required to understand how neuronal MDK is regulated relative to glioma growth, MDK has been previously implicated in the suppression of regulatory T cell (Treg) expansion^50^, as well as in the growth of experimental neurofibromas arising in the context of NF1^14,51^. While MDK activation of T cells is the operative mechanism underlying NF1-LGG pathogenesis, other etiologies are likely responsible for non-NF1 (sporadic) LGG initiation and growth. As such, elevated *MDK* expression was not observed in non-NF1 PA samples relative to normal brain tissue (Fig. 2i), and *MDK* expression is not correlated with LGG patient survival using available TCGA data (Supplementary Fig. 6f).

T cells normally provide a level of immune surveillance by trafficking from the cerebrospinal fluid through the brain, and draining back into the periphery via lymphatic vessels in the meningeal spaces^52^. Although not present in large numbers in the healthy brain, the amount of infiltrating T cells is increased in numerous different histologic brain tumor subtypes, each of which displays a unique pattern of immune cell infiltration and transcriptional signatures^53,54^. However, the contribution of CD4^+^ and CD8^+^ T cells to these tumors remains unclear. For example, pediatric LGG exhibit higher levels of T cell infiltration relative to their malignant counterparts^55^, while CD4^+^ and Foxp3^+^ T cell glioma content correlated with increased tumor progression^56^. Similarly, infiltrating T cells correlated with poor patient survival in one study^57^, whereas another demonstrated that CD8^+^ T cell content is associated with improved patient survival^58^.

Herein, we found that the majority of T cells in these low-grade murine gliomas are CD8^+^ lymphocytes, such that their depletion attenuated OPG growth in vivo. While these T cells are typically considered cytotoxic in nature, it is possible that they represent a population of exhausted T cells, which could be converted to tumoricidal^59^. Future experiments will be necessary to define their unique functional properties in this particular neoplastic setting. Similarly, whereas VLA-4 is required for CD8^+^ T cell infiltration into *Nf1* optic gliomas, as revealed by the anti-VLA4 antibody experiments, differential VLA-4 expression in CD4^+^ versus CD8^+^ T cells does not account for the preferential CD8^+^ T cell infiltration in *Nf1* murine optic gliomas^28^ and human NF1^54^ gliomas (Supplementary Fig. 6g, h). However, since T cells are recruited into murine *Nf1* optic gliomas by chemokines (Ccl2 and Ccl12) released by o-GSCs^27^, it is possible that differences in T cell population recruitment may result from differential responses to these chemokines. Future studies are focused on mechanistically defining how CD8^+ ^T cells are selectively recruited into these LGGs^27,60^.

As systemic immune regulators, T cells have the capability to educate macrophages/microglia in both health and disease^28,61^. In other brain diseases, infiltrating T cells are in close vicinity to influence microglia/macrophages^62^. In addition, T cells suppress microglia activation in the phase of inflammatory insults, as well as produce cytokines (e.g., IL-4) that can alter microglia function^63^. Similarly, in many types of cancer, T cells interact with tumor-associated microglia/macrophages (TAMs) through the secretion of factors, like IL-4 and IL-13^64^ and alter tumor growth or invasion. This connection is underscored by previous work from our laboratory in which microglia from athymic (*nu/nu*) mice exhibit reduced phagocytosis and reduced Ccl5 expression^12^. Consistent with T cell-mediated microglia education, incubating *nu/nu* mouse brain microglia with activated wild-type (WT) T cells or their CM resulted in restoration of normal Ccl5 levels. The ability of T cells to communicate with microglia to change their physiologic state and functional capabilities provides another mechanism to control brain homeostasis and influence CNS pathobiology. In light of the patient survival curves stratified by *CD8* and *CCL5* tumor expression (Figs. 6b, 8c, d), these T cell–microglia interactions may be particularly relevant to LGGs, where MDK/CCL4-independent mechanisms involving CD8^+^ T cells and microglia result in CCL5 support of tumor growth. Additional experiments using other LGG models will be required to extend these findings beyond NF1.

In summary, the neuron–immune–cancer axis elucidated in this study highlights the tightly choreographed interactions between neoplastic cells and their non-neoplastic neighbors (neurons, T cells, and microglia; Fig. 6h). Similar to other contexts in which numerous cell types (microglia, reactive astrocytes, and oligodendrocyte precursor cells) interact to impact on brain function (methotrexate-induced cognitive impairment^65^), the findings in this report expand the concept of the tumor microenvironment and the complex relationships between participating cell types, including neurons, but also offer opportunities to target the tumor ecosystem.

## Methods

### Mice

All mice, including *Nf1*^+*/*−^ mice (neomycin sequence insertion within exon 31 of the murine *Nf1* gene), *Nf1*^*flox/mut*^; GFAP-Cre (OPG) mice (*Nf1*^+*/−*^ mice with somatic *Nf1* gene inactivation in neuroglial progenitors at E15.5) and *Nf1*^*flox/flox*^ mice (controls)^66,67^, were maintained on a strict C57BL/6 background and used in accordance with an approved Animal Studies Committee protocol at Washington University. *Ccl5*^*−/−*^ mice were purchased from the Jackson Laboratory (JAX; 005090). Mice were housed in barrier facilities with controlled light–dark cycles (12:12 h) and ad libitum access to food and water.

### Anti-VLA4, anti-CD3, and anti-CD8 treatments

Six-week-old OPG mice (*n* = 10/cohort) were injected with 150 µg of anti-VLA4 (IgG2b), anti-CD3 (IgG2b), anti-CD8 (IgG2b), or rat IgG (isotype control, Bio X Cell) intraperitoneally every other day for 6 weeks. The brains and optic nerves were harvested when the mice reached 3 months of age, and the Iba1, Ki67, and CD3-positive cells analyzed by counting the number of cells showing a positive signal after immunohistochemistry^27^.

### Culture of mouse optic glioma stem cells, human iPSCs, and neurons

Optic nerves/chiasms were dissected from 3-month-old *Nf1* OPG mice and used to generate o-GSCs^68^. Single cell suspensions were obtained by digesting the optic nerve/chiasm in Trypsin Digest Medium, followed by maintenance in neural stem cell (NSC) medium (61% DMEM low glucose medium, 35% neurobasal medium, 2 mM l-glutamine, 1% penicillin/streptomycin; P/S) supplemented with Glutamax (200 mM), EGF (20 ng ml^−1^), FGF (20 ng ml^−1^), 1% N_2_, and 2% B27.

Isogenic human iPSCs homozygous or heterozygous for the c.2041C>T or c.6513T>A NF1 patient-specific *NF1* germline mutations were engineered using CRISPR/Cas9 technology by the Washington University Genome Engineering and iPSC Core Facility (GEiC). NPCs were differentiated by passaging iPSCs onto PLO/Laminin (Millipore Sigma)-coated plates using ReleSR (STEMCELL Technologies), followed by seeding at 200,000 cells cm^−2^ in NPC induction medium [50% DMEM F12 (Gibco), 50% Neurobasal medium (Gibco), supplemented with N2, B27 (Fisher), 2 mM GlutaMax (Gibco), 10 ng ml^−1^ hLIF, 4 µM CHIR99021, 3 µM SB431541, and 0.1 µM Compound E (all from STEMCELL Technologies)]. NPCs were maintained in this medium supplemented with 2 µM Dorsomorphin for 3 days, and without Dorsomorphin (STEMCELL technologies) for an additional 5 days. NPCs were subsequently incubated in NPC maturation medium (50% DMEM/F12, 50% Neurobasal medium supplemented with N2, B27, 2 mM GlutaMax, 10 ng ml^−1^ hLIF, 3 µM CHIR99021, and 2 µM SB431541), and were passaged weekly following Accutase (STEMCELL Technologies) dissociation according to manufacturer’s instructions. For differentiation into a mixed population of CNS neurons, NPCs were plated on PLO/Laminin-coated plates in Neuron Maturation Media (STEMCELL technologies) for 6 weeks prior to CM collection. CM was then used for T cell stimulation, MDK ELISA (LifeSpan Biosciences), CSF-2 (R&D Systems), and analysis using the Proteome Profiler Human Cytokine Array (R&D Systems ARY017).The collected neurons were used for mRNA isolation.

### Human PA samples

The PA specimens used for qRT-PCR analyses were obtained under an approved Human Studies Protocol as frozen tumor pellets from the St. Louis Children’s Hospital Pediatric Tumor Bank, and included nine NF1 patient PAs (3 males, 6 females; 10.11 ± 1.91 years), nine non-NF1 PAs (3 males, 6 females; 10.11 ± 1.76 years) and four non-neoplastic brain samples (2 males, 7 females; 10.25 ± 2.81 years). These de-identified specimens were used under an approved protocol from the Washington University School of Medicine Institutional Review Board. Human NF1-associated PA specimens used for immunohistochemistry were obtained from Dr. Sonika Dahiya under an approved protocol at the Washington University School of Medicine IRB. Immunohistochemistry was performed on adjacent paraffin sections using the antibodies listed in Supplementary Table 1. Detection was performed using the Vectastain Elite ABC kit (Vector Laboratories, Burlingame).

### Microglia/monocyte analyses

Each mouse brains were homogenized in the gentle MACS™ Dissociator (Miltenyi) with 5 ml of digestion solution (116 mM NaCl, 5.4 mM KCl, 26 mM NaHCO_3_, 1 mM NaH_2_PO_4_, 1.5 mM CaCl_2_, 1 mM MgSO_4_, 0.5 mM EDTA, 25 mM glucose, 1 mM cysteine, and 20 units ml^−1^ papain) for 30 min. 20 ml of PBS with 10% FBS was subsequently added to inactivate the digestion solution. Next, the cells were centrifuged (200×*g*, 20 min) in 20% Percoll overlaid with HBSS to remove the meninges. The cells were then treated with DNase, centrifuged and passed through a 70 µm strainer. The resulting cells maintained in minimal essential medium Earle’s medium supplemented with 1 mM l-glutamine, 1 mM sodium pyruvate, 0.6% d-(+)-glucose,1 ng ml^−1^ GM-CSF, 100 μg ml^−1^ P/S, 4% FBS, and 6% horse serum.

After 2 weeks, microglia were separated from the astrocytes by shaking (cultured in medium without GM-CSF 3 days before shaking, 200×*g*, 5 h, 37 °C). 5 × 10^5^ microglia/monocytes were grown in T cell CM for 24 h^12^, and Ccl5 measured by an ELISA kit (R&D Systems). Maraviroc (MCV) (Fisher Scientific, S2003), AZ0084 (MedChem Express, HY-119217) and Caffeic acid phenethyl ester (CAPE) (Fisher Scientific, 274310) were purchased from Fisher Scientific. The mouse recombinant cytokines [TNFα (Abcam, ab9740), GM-CSF (R&D Systems, 415ML101), Ccl2 (R&D Systems, 279-MC-010), Ccl1 (Fisher Scientific, EMCCL1),Ccl3 (Novus Biologicals, NBP2-35193), Ccl4 (R&D Systems, 451-MB-010), Ccl5 (Fisher Scientific, 478MR025), IL-1ra (Novus Biologicals, NBP2-35105), and Il-2 (R&D Systems, 402-ML-020)] were added to microglia and the Ccl5 levels quantified.

### Optic glioma stem cell proliferation and apoptosis assays

To measure proliferation, 10^4^ o-GSCs were seeded, and the total cell number counted 3 days later. To measure apoptosis, 10^5^ o-GSCs were plated into 24-well plates coated with poly-d-lysine (50 μg ml^−1^) and fibronectin (10 μg ml^−1^) in complete NSC medium. After 3 days, cells were grown in NSC medium without N2, B27, FGF, and EGF for 48 h, fixed in 4% paraformaldehyde (PFA), and apoptosis quantitated using a terminal deoxynucleotidyl transferase dUTP nick end labeling (TUNEL) kit (Sigma-Aldrich). MK2206 (Fisher Scientific, S1078) was purchased from Fisher Scientific.

### T cell analyses

WT or *Nf1*^+/−^ mouse spleens were homogenized into single cell suspensions by digesting in PBS containing 0.1% BSA and 0.6% Na-citrate, washed, and incubated with 120 Kunitz units of DNase I and red blood cell lysis buffer (eBioscience, 00433357). CD3^+^ or CD8^+^ T cells were isolated by negative selection using the pan-T-cell isolation kit II (Miltenyi Biotec, 130-095-130) or CD8a T Cell isolation kit (Miltenyi Biotec, 130-104-075), respectively. T cells were maintained at 2.5 × 10^6^ cells ml^−1^ in RPMI-1640 medium supplemented with 10% FBS and 1% P/S. CM from activated (1.25 µg ml^−1^ anti-mouse CD3 [Fisher Scientific, 16-0031-85] and 2 µg ml^−1^ anti-mouse CD28 [Fisher Scientific, 16-0281-82] antibody treatment for 2 days) and non-activated T cells (no antibody exposure) were collected, and the cytokines in the CM detected using the Proteome Profiler Mouse Cytokine Array Kit (R&D Systems, ARY006). Anti-MDK neutralizing antibody (Abcam, 170820) was used at a 1:50 dilution, while anti-Lrp1 blocking antibody (Fisher, MA1-27198) was used at 30 µg ml^−1^. Cyclosporin (C3662) and FK-506 (F4679) were purchased from Sigma-Aldrich. Mouse splenic T cells were plated at a density of 5 × 10^5^ cells in 96-well plates and incubated with 200 µl medium containing 30% iPSC-induced neuron CM and 70% complete RPMI-1640 (supplemented with 10% FBS and 1% P/S).

To assay migration, 5 × 10^5^ T cells were placed in the upper chamber of a transwell chamber (Corning, 6.5 mm insert, 3.0 µm polycarbonate membrane, CLS3398-2EA) with 200 µl serum-free PRIM 1640 media. 500 µl of chemoattractant media (MDK or CSF-2, concentrations indicated in figures) was added to the lower chamber, and the number of T cells in the lower chambers were counted 4 h later.

### Flow cytometry

Spleens from anti-CD3-treated mice were harvested when the mice reached 3 months of age, and single-cell suspensions without red blood cells were prepared from mouse spleens. Cells were incubated with anti­-CD3­PB antibodies (BioLegend, 100431) at 4 °C for 30 min. Spleens from anti-CD8-treated mice were harvested when the mice reached 3 months of age, and single-cell suspensions without red blood cells were prepared from mouse spleens. Anti­-CD4­-PerCP antibodies (BioLegend, 100431) and anti-CD8-APC antibodies (BioLegend, 100713) were added, and incubated with cells at 4 °C for 30 min. Flow cytometry was performed using an Attune NxT Flow Cytometer (Thermo Fisher Scientific), and analyzed using FlowJo v10.6.1 software.

### Immunohistochemistry and immunofluorescence

Mice were euthanized and perfused transcardially with Ringer’s solution, followed by 4% PFA, and the brains and optic nerves/chiasms were processed for paraffin embedding and sectioning. Immunohistochemistry was performed on adjacent paraffin sections with antibodies listed in Supplementary Table 1. Detection was performed using the Vectastain Elite ABC kit (Vector Laboratories, Burlingame). All sections were photographed with a digital camera (Optronics) attached to an inverted microscope (Nikon). For leptomeningeal analyses, 6-week old OPG and control mice were perfused with Ringer’s solution, followed by PFA, and storage in 30% sucrose. Floating sections of brains (20 µm-thick sections) were stained with anti-CD3/CD4/CD8 antibodies for 1 h at 4 °C after 0.1% PBS-T (Triton X-100) treatment (5 min) and corresponding secondary antibodies (Alexa-fluor 488 for CD3 and 568 for CD4/CD8). DAPI was used for counterstaining during mounting.

### Western blotting

Nuclear protein extracts were obtained using RIPA and protein concentration was determined using the Pierce BCA protein assay kit. Up to 50 µg of protein per extract were separated in SDS–polyacrylamide gels by electrophoresis. After protein transfer onto PVDF membrane, the membranes were incubated with the indicated antibodies. Antibody binding was detected after incubation with a secondary antibody coupled to IRDye. The blots were analyzed using Image Studio Lite Ver 5.2 software. Images of the original immunoblots can be found in Supplementary Figs. 8 and 9.

### RNA extraction and real-time qRT–PCR

RNA was isolated from cells using the RNeasy kit (QIAGEN), while RNA was isolated from mouse optic nerves using the Trizol reagent (Life Technologies). Real-time qRT–PCR was performed using the specific primers listed in Supplementary Table 2. ΔΔCT values were calculated using *H3f3a* as an internal control.

### Survival curves

Kaplan–Meier survival curves were obtained from the MSKCC computational biology cancer genomics portal (http://www.cbioportal.org)^30,69^, which contains two annotated TCGA datasets (Brain Lower Grade Glioma (TCGA, Provisional) and Brain Lower Grade Glioma (TCGA, PanCancer Atlas). Statistical significance was set at *P* < 0.05.

### Lovastatin treatment

100,000 human iPSC-derived NPCs were plated on PLO/laminin-coated six-well plates, allowed to differentiate into neurons in neuron maturation media for 6 weeks, and were then treated with varying concentrations of lovastatin for 24 h. After an additional 12 h, the conditioned media was collected for midkine ELISA measurements.

### Primary neonatal microglia isolation and culture

Mouse pups (postnatal day 0–4; PN0–4) were euthanized, and their brains (without the cerebellum and olfactory bulb) were collected and stripped of the overlying meninges. The brains were then washed twice with ice-cold wash buffer and incubated with 3 ml of trypsin (2.5%) at 37 °C for 20 min, followed by inactivating trypsin in 4 mls of complete DMEM culture media (with 10% FBS). The cells were then treated with DNase, resuspended in 10 mls of DMEM complete media, passed through a 70 µm strainer, and seeded in flasks. The culture media was changed on day 3, and cells were fed with an additional 3 mls of fresh complete DMEM on day 7. Between days 8 and 14, microglia were isolated by shaking the flasks for 20–25 min at room temperature on a shaker at 250 rpm.

### Splenic monocyte isolation

For monocyte isolation, spleen single cell suspensions were incubated with CD11b-specific magnetic beads, and captured using a magnetic separator. 5 × 10^5^ microglia/monocytes were grown in T cell CM for 24 h^1^, and Ccl5 measured by an ELISA kit (R&D Systems).

### Statistical analyses

Data analyses were performed using GraphPad Prism. Unpaired two-tailed Student’s *t*-tests were used to determine differences between two groups. Multiple comparisons were analyzed by one-way analysis of variance (ANOVA) with Dunnett’s multiple comparisons test. Statistical significance was set at *P* < 0.05. All data are presented as mean values ± SEM. All experiments have been replicated at least three independent times with similar results.

## Supplementary information

Supplementary Information

## Data Availability

The data are available within the Article, Supplementary Information and available from the authors upon request. The source data for Figs. 1–8 and Supplementary Figs. 1–9 are provided as a Source Data file. Additional data were extracted from the Memorial Sloan Kettering Cancer Center cBioPortal for Cancer Genomics (https://www.cbioportal.org) and the Immunological Genome Project (www.immgen.org).
