# Supplementary material for: Midkine activation of CD8+ T cells establishes a neuron–immune–cancer axis responsible for low-grade glioma growth
Source: Nat Commun. 2020 May 1;11:2177. doi: 10.1038/s41467-020-15770-3 (PMC7195398; doi:10.1038/s41467-020-15770-3)
Supplement: Supplementary file 1 — Supplementary Information [file 41467_2020_15770_MOESM1_ESM.pdf]

Supplementary Materials

Midkine activation of CD8⁺ T cells establishes a neuron-immune-cancer axis responsible for low-grade glioma growth

Guo X, Pan Y, Xiong M, Sanapala S, Anastasaki C, Cobb O, Dahiya S, Gutmann DH

Address correspondence to: David H. Gutmann, MD, PhD, Department of Neurology, Washington University, Box 8111, 660 S. Euclid Avenue, St. Louis, MO, USA 63110. 314-362-7379 (phone); gutmannd@wustl.edu (email); ORCID #0000-0002-3127-5045

Supplementary Figures

Supplementary Figure 1

Supplementary Figure 2

Supplementary Figure 3

Supplementary Figure 4

Supplementary Figure 5

Supplementary Figure 6

Supplementary Figure 7

Supplementary Figure 8

Supplementary Figure 9

Supplementary Tables

Supplementary Table 1

Supplementary Table 2

Supplementary Figures

Supplementary Figure 1

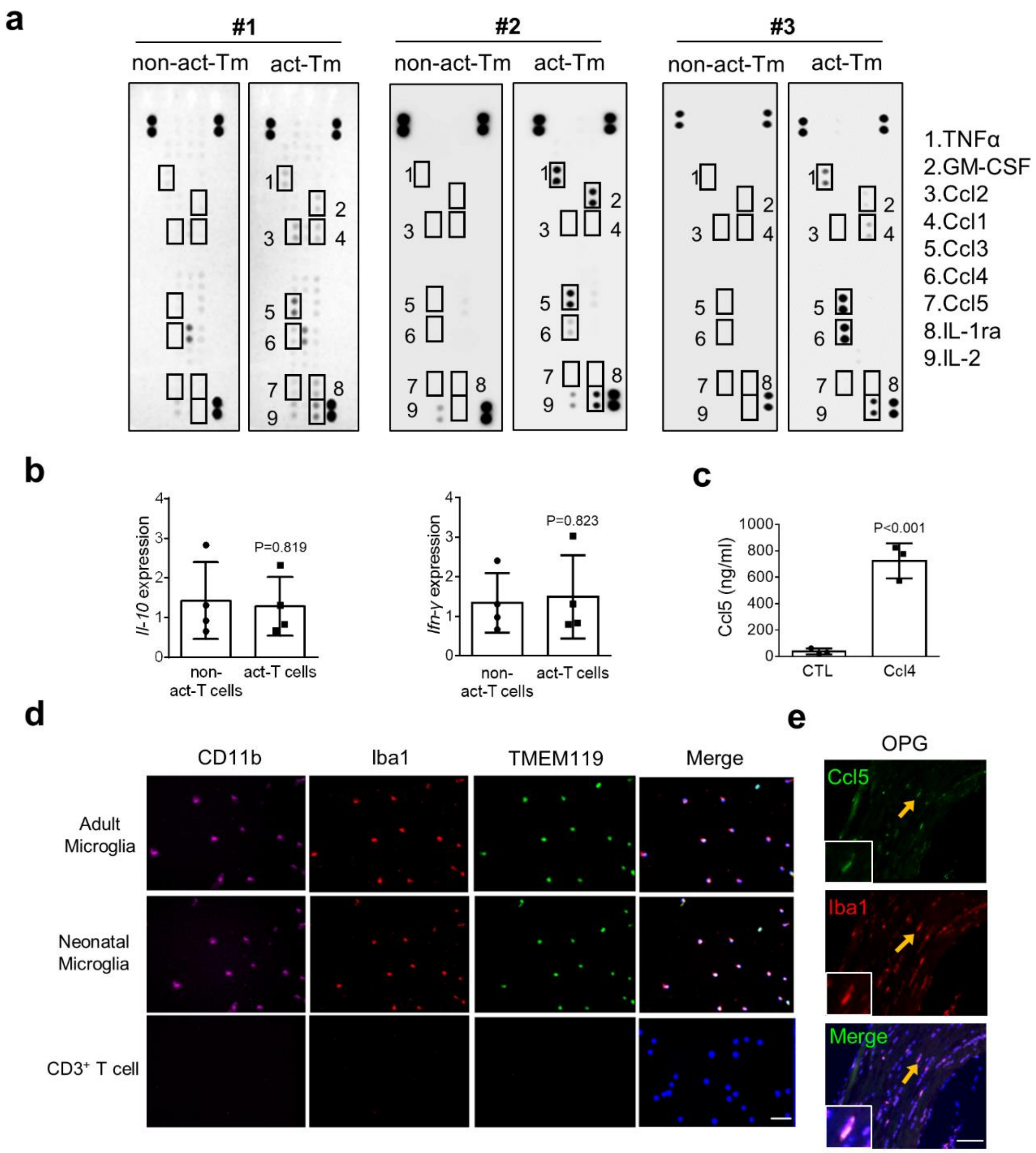

Supplementary Figure 1. **Neonatal microglia respond similarly as adult microglia to Ccl4.** (**a**) WT splenic T cells were seeded at 2.5x10^{6} cells/ml in complete PRIM1640 medium, followed by two days CD3/CD28 stimulation (activated; act-Tm) or vehicle (PBS) treatment (non-

activated; non-act-Tm). CM was collected for chemokine array analysis using three independently treated sample sets. (**b**) Two cytokines (IL-10 and IFN-γ) whose CM expression was not different in the chemokine array between activated versus non-activated T cell CM exhibited no change at the RNA level using qRT-PCR. (**c**) WT neonatal microglia were stimulated with Ccl4 (6000 pg/ml) for 24 h at the concentrations detected in the activated T cell CM, and Ccl5 examined by ELISA. (**d**) The expression of CD11b, Iba1 and TMEM119 on adult microglia, neonatal microglia, and $CD3^+$ T cells (negative control) were examined by immunofluorescence. (**e**) Double-labeling immunofluorescence staining reveals Ccl5 staining in $Iba1^+$ cells. (**d-e**) Scale bars, 40 µm. All data are presented as the mean ± SEM. (**b, c**) Two-tailed Student's t-test. (**a**) n=3 independent biological samples were examined over 3 independent experiments. (**b**) The bar graphs represent means ± SEM of (**b**) n=4 independent biological samples. (**c**) This representative experiment was conducted with n=3 independent biological samples and was replicated two additional times with similar results. (**d**, **e**) These are representative images of n=3 independent biological samples examined with similar results. Exact P values are indicated within each panel; N.S.; not significant. From left to right in each panel: (**b**) P=0.819, P=0.823; (**c**) P<0.001.

Supplementary Figure 2

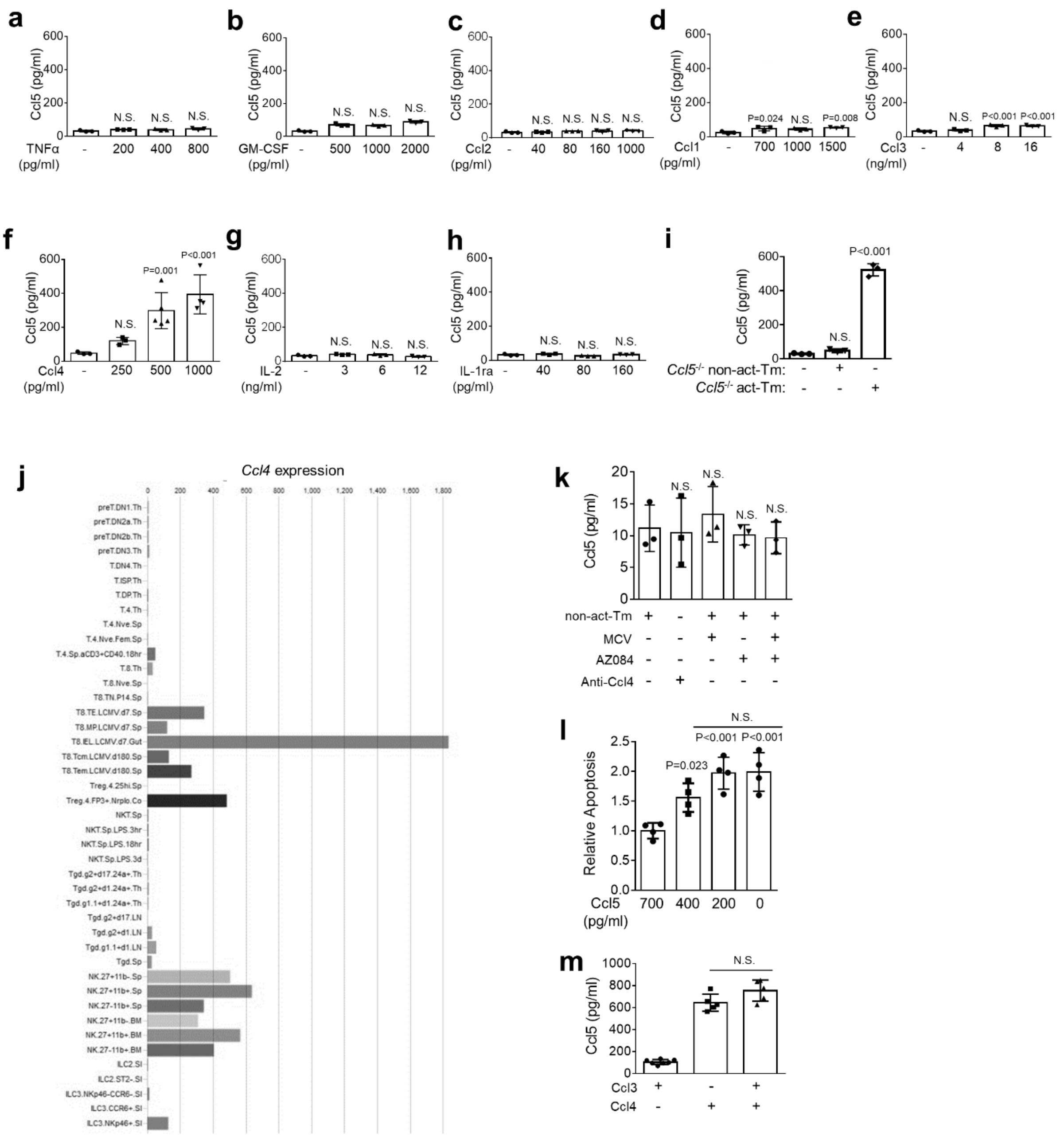

Supplementary Figure 2. Ccl4 increases microglial Ccl5 production. (**a-h**) The ability of the candidate cytokines detected in activated T cell CM to induce microglial Ccl5 release (ELISA) was measured over a concentration range. Ccl3 (8ng/ml and 16ng/ml) increased microglial Ccl5 production by 2-fold, whereas Ccl4 increased microglial Ccl5 production by 6.3 and 8.5-fold at

500pg/ml and 1ng/ml, respectively. (**i**) Activated *Ccl5-/-* T cell CM exhibited a similar potency as activated WT T cell CM in inducing microglia Ccl5 production. (**j**) *Ccl4* mRNA expression in different immune system cell types using the Immunological Genome Project on-line database (www.immgen.org; supported by the National Cancer Institute)[2]. (**k**) Anti-Ccl4, MCV and AZ084 treatments did not change microglia Ccl5 production following exposure to non-activated T cell conditioned medium (non-act-Tm). (**l**) The effect of reducing Ccl5 on o-GSC apoptosis over a dose range was examined. (**m**) The combination of Ccl3 (8000 pg/ml) and Ccl4 (6000 pg/ml) did not exhibit further induction of microglial Ccl5 relative to Ccl4 (6000 pg/ml) alone. All data are presented as the mean ± SEM. (**a-i**, **k-m**) One-way ANOVA with Bonferroni post-test correction. (**a-e**) Bar graphs represent the means ±SEM of n=3 independent biological samples. (**f**, **g-i**) This representative experiment was conducted with (**f**) control and Ccl4 250 pg ml^{-1} groups n=3, Ccl4 500 and 1000 pg ml^{-1} groups n=4, (**g-i**) n=3, (**k-m**) n=4 independent biological samples and were replicated two additional times with similar results. Exact P values are indicated within each panel; N.S.; not significant. From left to right in each panel: (**a**) all N.S.; (**b**) all N.S.; (**c**) all N.S.; (**d**) P=0.024, N.S., P=0.008; (**e**) N.S., P<0.001, P<0.001; (**f**) N.S., P=0.001, P<0.001; (**g**) all N.S.; (**h**) all N.S.; (**i**) N.S., P<0.001; (**k**) all N.S.; (**l**) P=0.023, P<0.001, P<0.001, upper comparison, N.S.; (**m**) N.S.

Supplementary Figure 3

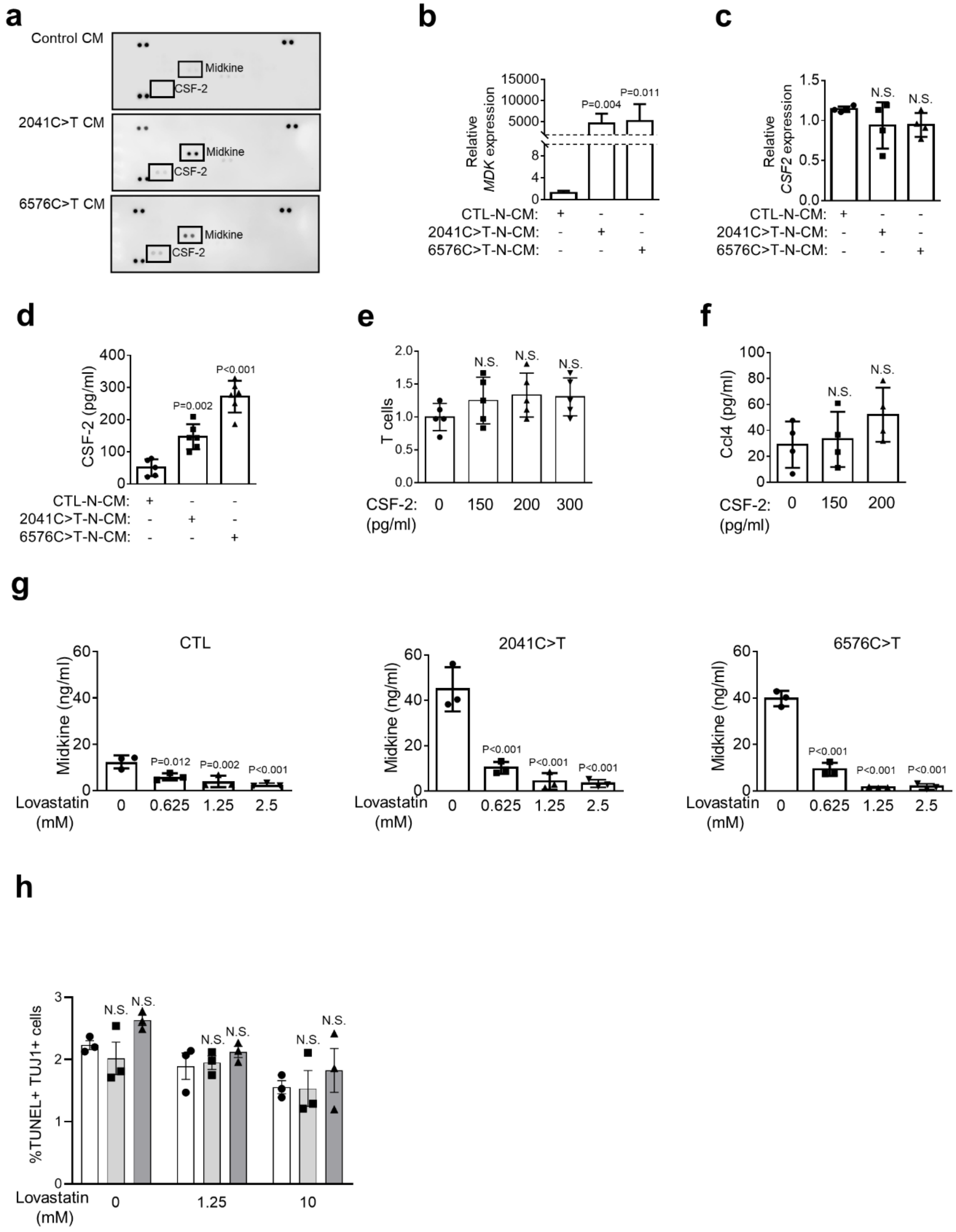

Supplementary Figure 3. MDK expression is increased in *NF1*-mutant neuron conditioned medium. (**a**) Human iPSC-induced neurons harboring homozygous NF1 patient germline *NF1* gene mutations (c.2041C>T and c.6576C>T) released higher levels of midkine (MDK) and CSF-2 compared to WT iPSC-induced neurons. (**b**) Quantitative real-time RT-PCR reveals increased *MDK* gene expression in hiPSC-induced neurons harboring homozygous NF1 patient germline *NF1* gene mutations (c.2041C>T and c.6576C>T). (**c**) *CSF2* RNA expression was not changed in c.2041C>T or c.6576C>T *NF1*-mutant, relative to control, hiPSC-derived neurons. (**d**) CSF-2 protein expression was increased in CM from c.2041C>T or c.6576C>T *NF1*-mutant, relative to control, hiPSC-derived neurons. (**e**) No change in T cell migration was observed in response to various CSF-2 concentrations. (**f**) No change in CD3$^+$ T cell Ccl4 expression was observed following CSF-2 exposure. (**g**) Control (CTL), c.2041C>T mutant and c.6576C>T mutant hiPSC-derived neurons were treated with lovastatin over a dose range (0.625 to 2.5 mM), and midkine release in the CM was measured. (**h**) Lovastatin did not affect *NF1*-het hiPSC-derived neuron apoptosis (%TUNEL$^+$ cells). White bars, control hiPSC-derived neurons; light grey bars, c.2041C>T hiPSC-derived neurons; dark grey bars, c.6576C>T mutant hiPSC-derived neurons. All data are presented as the mean ± SEM. (**b-h**) One-way ANOVA with Bonferroni post-test correction. (**b-c**) Bar graphs represent the means ±SEM of (**b**) n=5 or (**c**) n=4 independent biological samples. (**d-g**) These representative experiments were conducted with (**d**, **e**) n=5, (**f**) n=4, (**g, h**) n=3 independent biological samples and were replicated two additional times with similar results. Exact P values are indicated within each panel; N.S.; not significant. From left to right in each panel: (**b**) P=0.004, P=0.011; (**c**) both N.S.; (**d**) P=0.002, P<0.001; (**e**) all N.S.; (**f**) both N.S.; (**g**) left P=0.012, P=0.002, P<0.001; middle P<0.001, P<0.001, P<0.001; right P<0.001, P<0.001, P<0.001; (**h**) all N.S..

Supplementary Figure 4

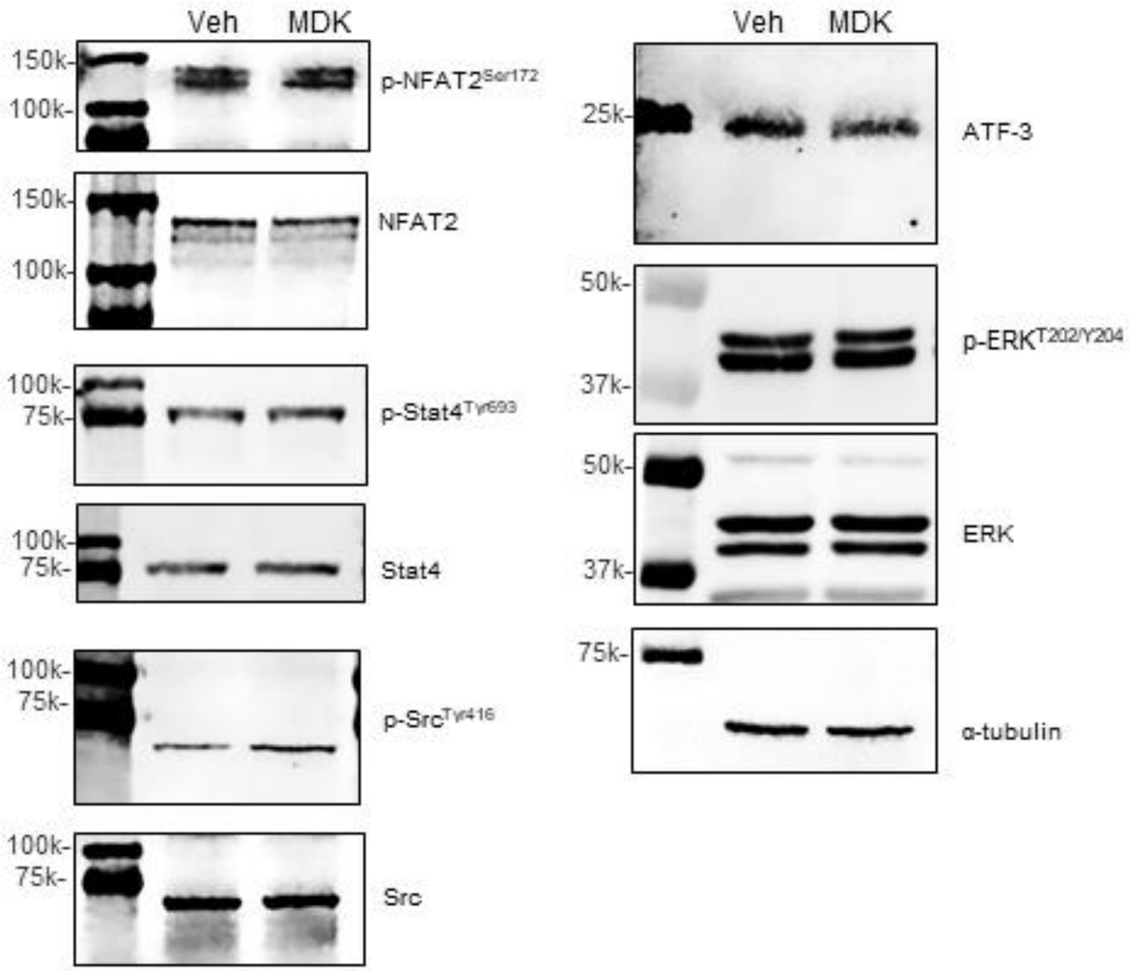

Supplementary Figure 4. Immunoblotting of different proteins expression in T cells after midkine treatment. No differences in NFAT2, Stat4, Src, ATF-3 and ERK activation were observed following midkine (50 ng/ml) treatment of T cells, as determined by immunoblotting with phospho-specific antibodies. n=3 independent biological samples were examined over 3 independent experiments with similar results. Molecular weight markers are denoted at the left side of each blot.

Supplementary Figure 5

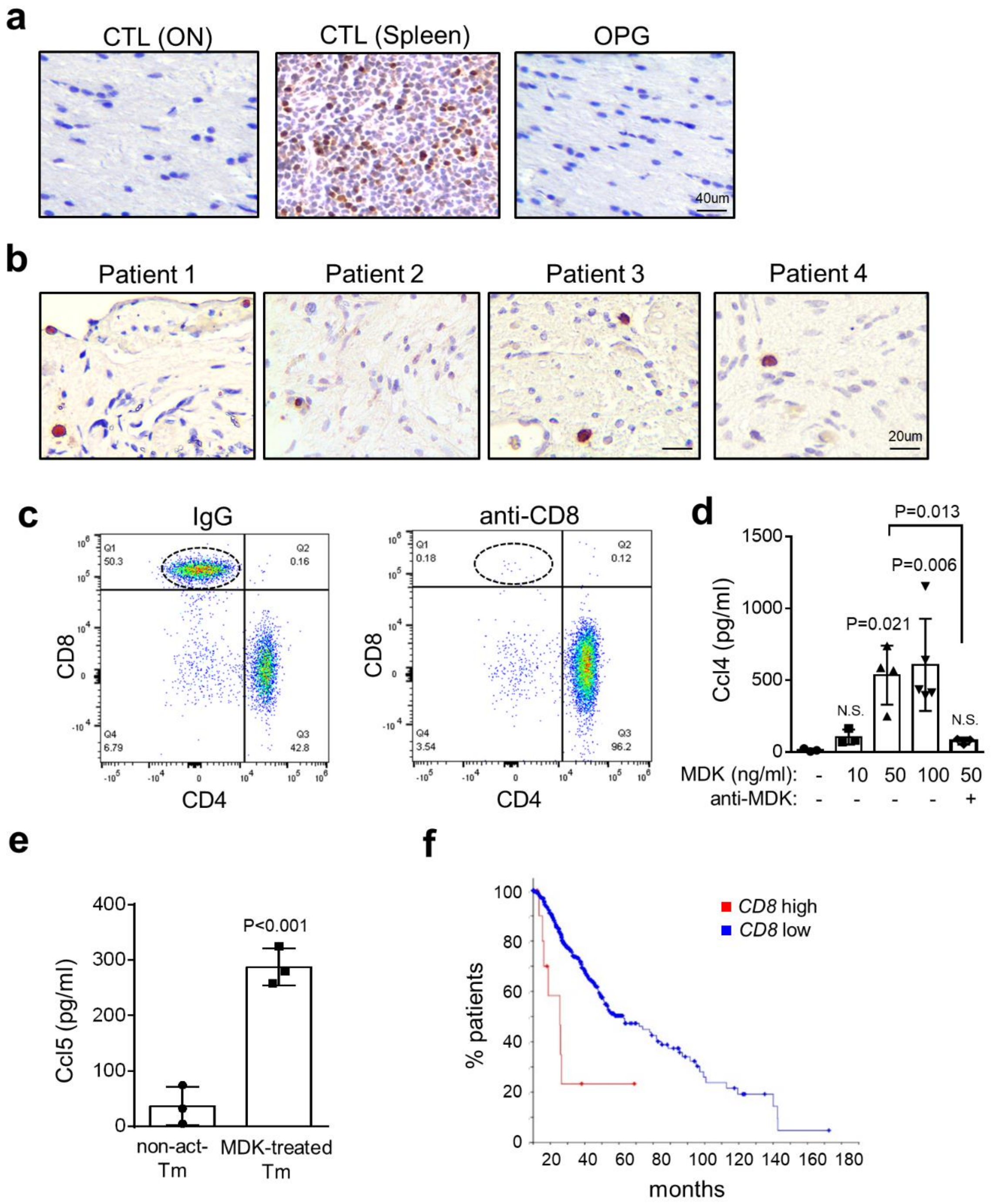

Supplementary Figure 5. CD8^{+} T cells mediate MDK/Ccl4-induced microglial Ccl5 production. (**a**) No Foxp3^{+} cells were detected in the optic nerves (ON) of control (CTL) or optic glioma (OPG)-bearing mice. WT mice spleen was used as a positive control for

immunohistochemistry. Scale bar, 40 µm. (**b**) CD8$^+$ T cells in PA specimens from NF1 patients (N=4 independent patient tumors). Scale bars, 20 µm. (**c**) CD8$^+$ T cells were depleted in splenocytes from anti-CD8 treated mice (0.18%), compared to IgG treated mice (50.3%), as measured by flow cytometry. (**d**) MDK (50 ng/ml or 100 ng/ml) stimulation for 48 h increased CD8$^+$ T cell Ccl4 production. Anti-MDK antibodies reduced MDK-induced Ccl4 production by CD8$^+$ T cells. (**e**) MDK-activated (50 ng/ml) CD8$^+$ T cell CM (MDK-treated Tm) increased microglial Ccl5 production relative to non-activated CD8$^+$ T cell CM (non-act-Tm). (**f**) Kaplan-Meier analysis reveals reduced disease/progression-free survival time in patients with low-grade gliomas harboring high *CD8* expression (P=3.678e-4). Data were obtained from the MSKCC computational biology cancer genomics portal (http://www.cbioportal.org), which contains annotated TCGA data (Brain Lower Grade Glioma (TCGA, Provisional; mRNA expression Z score=2, P<0.001). (**d, e**) Bar graphs represent the means ± SEM of (**d**) n=4 and (**e**) n=3 independent biological samples. One-way ANOVA with Bonferroni post-test correction. Exact P values are indicated within each panel; N.S.; not significant. From left to right in each panel: (**d**) N.S., P=0.021, P=0.006, N.S.; upper comparison, P=0.013; (**e**) P<0.001.

Supplementary Figure 6

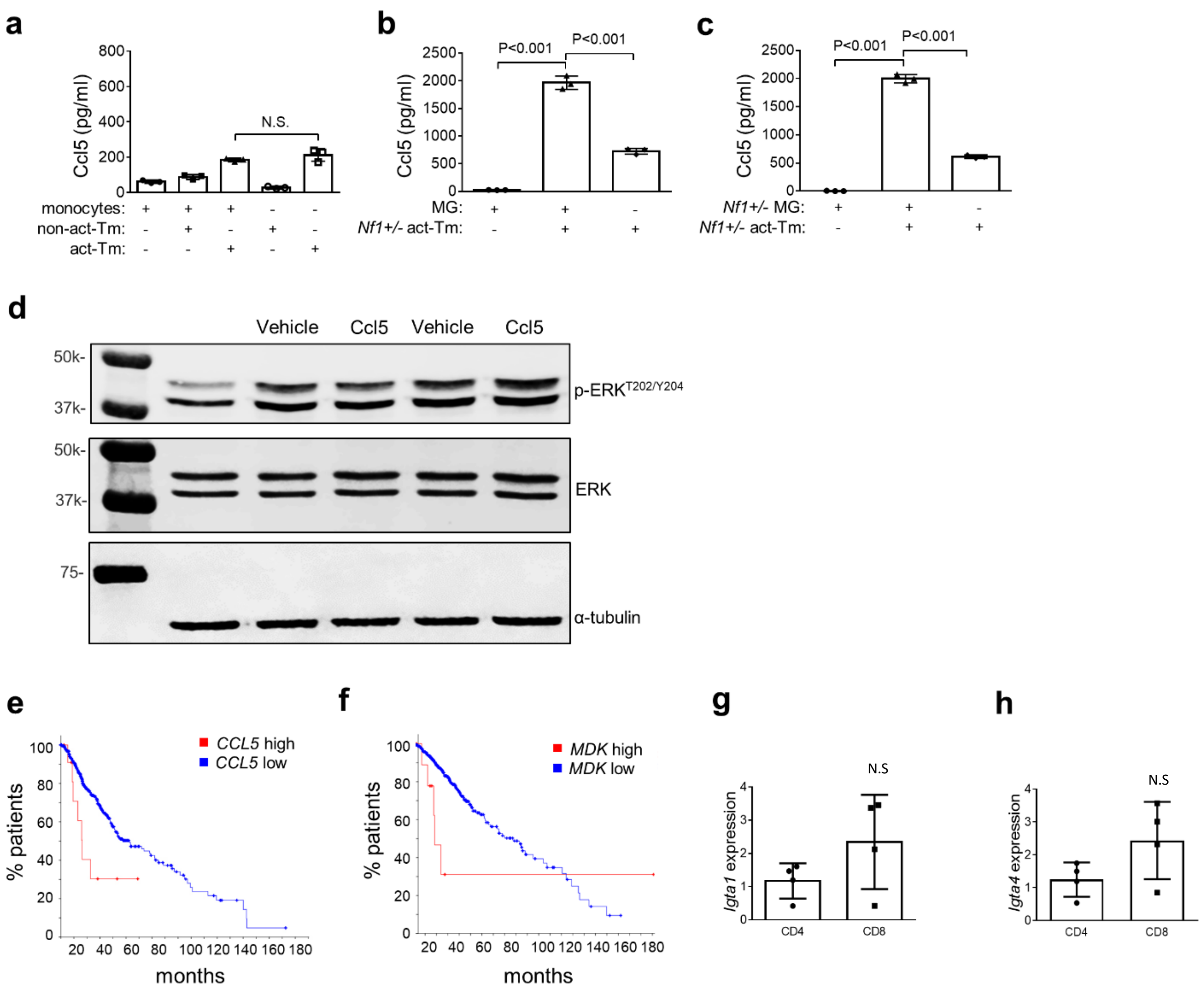

Supplementary Figure 6. CCL5 is a critical regulator of low-grade glioma growth. (**a**) Activated T cell CM (act-Tm) did not induce WT peripheral splenic monocytes (macrophages) to produce Ccl5. Naïve T cell CM (non-act-Tm) was included as a control. Activated *Nf1*+/- T cell CM treatment increases (**b**) WT and (**c**) *Nf1*+/- microglial (MG) Ccl5 production, similar to that observed following exposure to activated WT T cell CM. (**d**) Ccl5 stimulation did not increase ERK activation (p-ERK$^{Thr202/Tyr204}$) in o-GSCs by immunoblotting. Total ERK and α-tubulin served as internal protein loading controls. (**e**) Kaplan-Meier analysis demonstrates shorter

disease/progression-free survival time in patients with low-grade gliomas harboring high *CCL5* expression (P=0.0154). Data were obtained from the MSKCC computational biology cancer genomics portal (http://www.cbioportal.org), which contains annotated TCGA data (Brain Lower Grade Glioma [TCGA Provisional], mRNA expression Z score=2, P<0.001). (**f**) Kaplan-Meier analysis of low-grade glioma patients harboring high and low *MDK* expression (P= 0.181). Data was obtained from the MSKCC computational biology cancer genomics portal (http://www.cbioportal.org), which contains annotated TCGA data (Brain Lower Grade Glioma (TCGA, Firehose Legacy), mRNA expression Z score=2. (**g-h**) RT-qPCR of *Igta1* and *Igta4* in $CD4^+$ and $CD8^+$ T cells. All data are presented as the mean ± SEM. (**a-c**) One-way ANOVA with Bonferroni post-test correction, (**g, h**) Two-tailed Student's t-test. (**a-c**) These representative experiments were conducted with n=3 independent biological samples and were replicated two additional times with similar results. (**g, h**) Bar graphs represent means ± SEM of n=4 independent biological samples. Exact P values are indicated within each panel; N.S.; not significant. From left to right in each panel: (**a**) N.S., (**b**) P<0.001, P<0.001; (**c**) P<0.001, P<0.001; (**g**) N.S.; (**h**) N.S. (**d**) These are representative images of n=3 independent biological samples examined over three independent experiments with similar results. Molecular weight markers are denoted at the left side of each blot.

Supplementary Figure 7

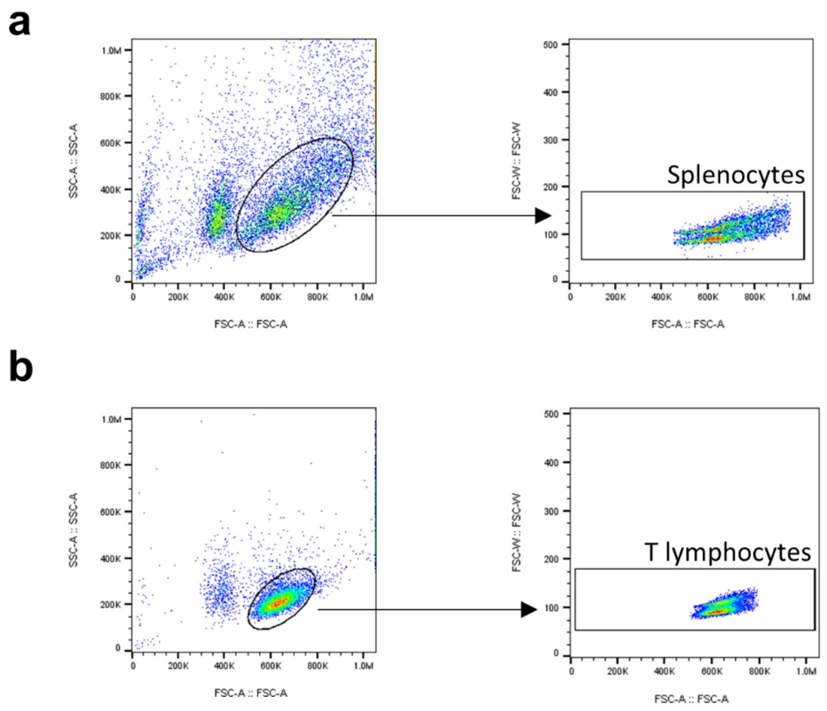

Supplementary Figure 7. Gating strategies used for cell sorting. (**a**) Gating strategy to sort splenocytes in **Fig. 4a**. (**b**) Gating strategy to sort T lymphocytes in **Supplementary Figure 5c** (T cells were pre-sorted from splenocytes using pan-T-cell isolation kit as described in “Methods” before the FACS analysis).

Supplementary Figure 8

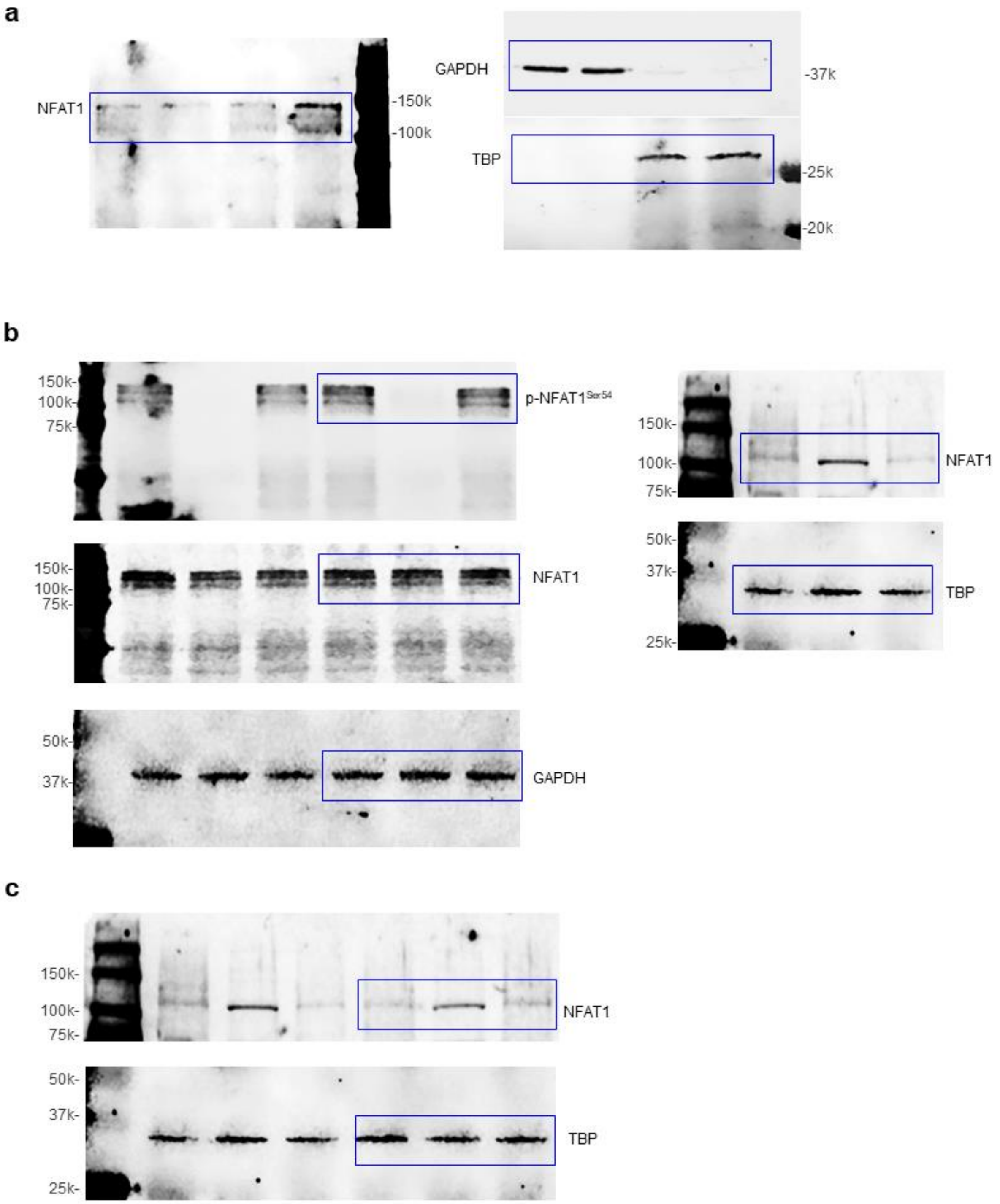

Supplementary Figure 8. Original western blots for Figure 3. Original uncropped immunoblots referring to **Figures 3d** (**a**), **3e** (**b**) and **3f** (**c**).

Supplementary Figure 9

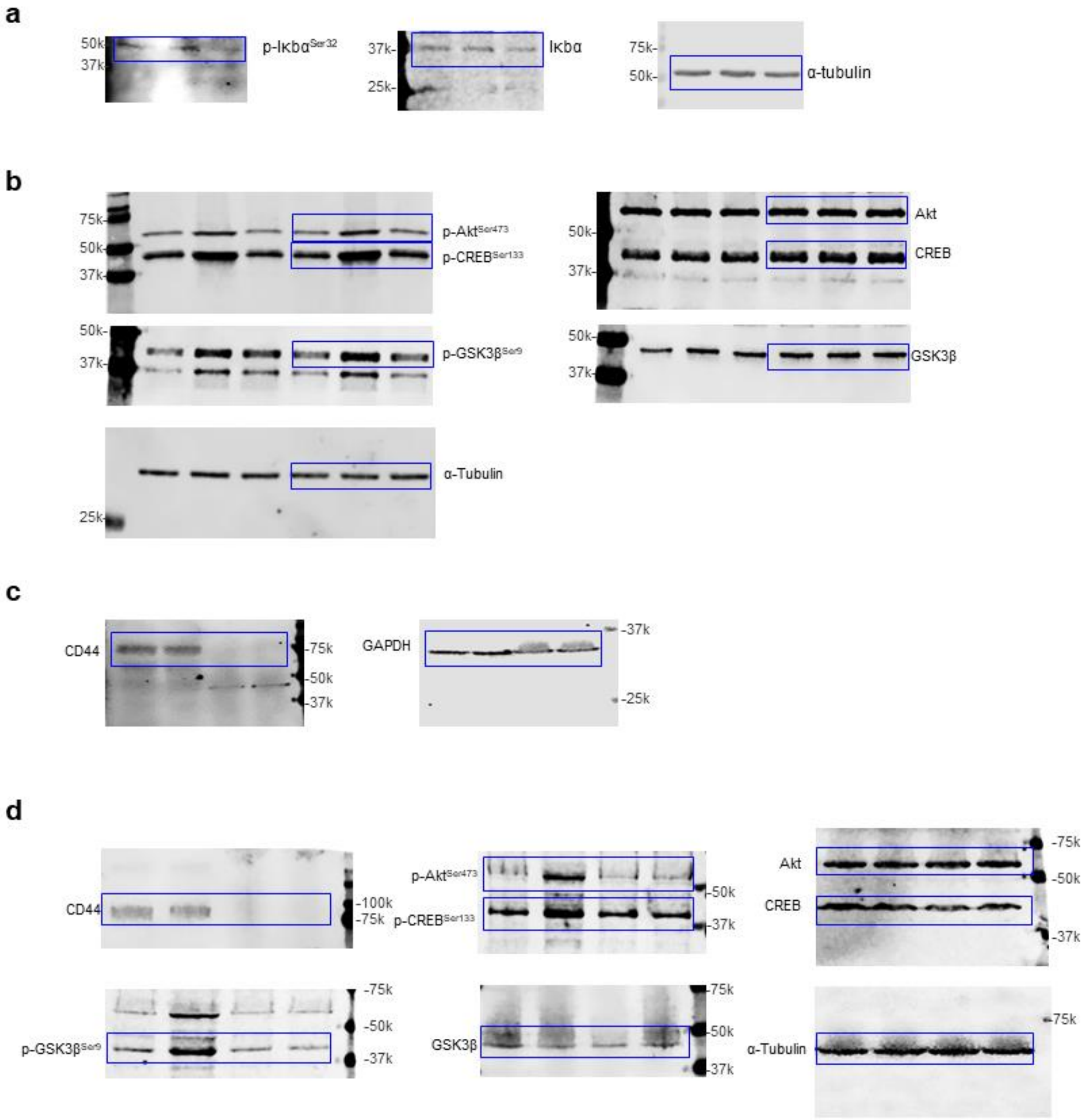

Supplementary Figure 9. Original western blots for Figure 7. Original uncropped immunoblots referring to **Figures 7a** (**a**), **7e** (**b**) and **7h** (**c**) and **7j** (**d**).

Supplementary Table 1. Antibodies used.

Antibody	Host	Source	Lot/Clone	Dilution
Akt (WB)	rabbit	Cell Signaling, 9272	28	1:1000
anti-goat Alexa 488 (IF)	donkey	Thermo, A-11055	2051233	1:200
ATF-3 (WB)	rabbit	Cell Signaling, 3393	1	1:1000
Biotinylated anti-mouse IgG (IHC)	goat	Vector, BA-9200	ZE1207	1:200
Biotinylated anti-rabbit IgG (IHC)	goat	Vector, BA-1000	ZF0809	1:200
Biotinylated anti-rat IgG (IHC)	goat	Vector, BA-4000	ZB1216	1:200
Ccl5 (IF, IHC)	rabbit	Abcam, ab9679	GR5419-73	2 µg/ml
Ccl4 (IHC)	rabbit	AVIVA Sys., OAAB00178	SA101014	1:50
CD3 (IHC/IF, mouse)	rat	Abcam, 11089	GR3210117-1	1:50
CD3-PB (FACS)	rat	BioLegend, 100213	B189839	1.25 µg/ml
CD4 (IF)	rabbit	Abcam, 133616	GR3240246-1	1:100
CD4-PerCP (FACS)	rat	BioLegend, 100431	GR3240246-1	1.25 µg/ml
CD8a (IF)	rat	Abcam, 22378	GR3237266-1	1:100
CD8a (IHC)	rabbit	Cell Signaling, 98941	8	1:600
CD8a-APC/Cy7 (FACS)	rat	BioLegend, 100713	ZB13216	2.5 µg/ml
CD11b (IF)	rat	Abcam, 8878	GR3306578-1	2 µg/ml
CD44 (WB)	rabbit	Abcam, 157107	GR3210501-1	1:2000
CREB (WB)	rabbit	Cell Signaling, 9197	16	1:1000
Erk1/2 (WB)	rabbit	Cell Signaling, 9102	27	1:1000
Foxp3 (IHC)	mouse	Santa Cruz sc-53876	A2918	1:1000
GAPDH (WB)	mouse	Abcam, 8245	GR3395417-1	1:1000
Gsk-3β (WB)	rabbit	Cell Signaling, 12456	8	1:1000
Iba1 (IHC, IF)	goat	Abcam, 5076	GR3295070-1	2 µg/ml

Iba1 (IF)	rabbit	Wako, 019-19741	CAM6570	1:1000
Ki67 (IHC)	mouse	BD Pharmingen, 550609	8239549	1:500
Midkine (IHC)	rabbit	Abcam, 170820	GR169231-16	1:50
NFAT1 (WB)	rabbit	Cell Signaling, 5558	4	1:500
NFAT2 (WB)	rabbit	Cell Signaling, 8023	6	1:1000
Phospho-CREB (Ser133) (WB)	rabbit	Cell Signaling, 9199	14	1:1000
Phospho-CREB (Ser133) (IHC)	rabbit	Abcam, 32096	GR3231215-4	1:200
Phospho-Erk1(Thr204)/Erk2(Tyr187) (WB)	rabbit	Cell Signaling, 5726	1	1:1000
Phospho-Gsk-3β (Ser9) (WB)	rabbit	Cell Signaling, 5558	9	1:1000
Phospho-Akt (Ser473) (WB)	rabbit	Cell Signaling, 4060	27	1:1000
Phospho-Akt (Ser473) (IHC)	rabbit	Abcam, 81283	GR3241584-5	1:100
Phospho-NFAT1 (Ser54) (WB)	rabbit	Invitrogen, 44-99G	2020147	1:300
Phospho-NFAT2 (Ser172) (WB)	mouse	Novus, 679340	MAP540-SP	1:500
Phospho-Src (Tyr416) (WB)	rabbit	Cell Signaling, 6943	4	1:1000
Phospho-Stat4 (Tyr693) (WB)	rabbit	Abcam, 28815	28815	1:500
Src (WB)	rabbit	Cell Signaling, 2109	7	1:1000
Stat4 (WB)	rabbit	Cell Signaling, 2653	3	1:1000
TBP (WB)	mouse	Abcam, 818	GR3261953	1:500
TMEM119 (IF)	rabbit	Abcam, 209064	UJ2867641	1 µg/ml
α-tubulin (WB)	mouse	Cell Signaling, 3873S	15	1:10,000

WB, Western blot; IHC, immunohistochemistry; IF, immunofluorescence; HRP, horseradish peroxidase

Supplementary Table 2. qRT-PCR primers used.

**Gene**	**Forward primer sequence**	**Reverse primer sequence**
Alk (mouse)	CATTGATCCTCTCCGTCGTG	CAGTTCCATCTGCATAGCCT
ACTB (human)	TGAAGTGTGACGTGGACATC	GGAGGAGCAATGATCTTGAT
Ccl4 (mouse)	CCACTTCCTGCTGTTTCTCTTA	CTGTCTGCCTCTTTTGGTCAG
Ccl5 (mouse)	AATCTTGCAGTCGTGTTTGTCA	AGCTCATCTCCAAATAGTTGATGT
Ccr5 (mouse)	TGTCTTCATGTTAGATTTGTACAGC	GTGCTGACATACCATAATCGATG
Ccr8 (mouse)	CTCAGAAGAAAGGTCGCT	GAGGAACTCTGCGTCACAG
Cd44 (mouse)	GGATGAATCCTCGGAATTACCA	GCTTTCAACAGTACCTTACCA
Cspg5 (mouse)	CATGATGACTGTGTTCTTTGCC	GTCGTTGTGGAGCTCAGATG
H3f3a (mouse)	CGTGAAATCAGACGCTATCAGAA	TCGCACCAGACGCTGAAAG
Ifng (mouse)	CTGAGCAATGAACGCTACACA	TCCACATCTATGCCACTTGAG
Il10 (mouse)	GTCATCGATTTCTCCCCTGTG	ATGGCCTTGTAGACACCTTG
Igta1 (mouse)	CATCCCTCATAACACCACCTT	CCAGCGATATAGAGCACATCT
Igta4 (mouse)	GATGGCTTCTCAGATCTCCTTG	CCCTTTCCATTTCAACCATCAC
Lratp1 (mouse)	CATACCATCAACATCTCCCTCA	CCGTTTCGGTTACAGACAAAG
Lrp6 (mouse)	TGACATCCATGCAGTAAAGGAG	GAGCATCTTGTCGTACCATCT
Mdk (mouse)	AGGCTTCTTCCTTCTCGCCCTTCTT	GGCTTTGGTCTTTGACTTGGTCTTG
MDK (human)	GTCTGAGCTGCGTCCTG	GCCCTTCTTCACCTTATCTTTC
Ptprz1 (mouse)	TTCTTAAAGCACATTCGTTCTCAA	TCCGTTTCCTTGCTGAGTATG

Supplementary References

1. Pan Y*, et al.* Athymic mice reveal a requirement for T-cell-microglia interactions in establishing a microenvironment supportive of *Nf1* low-grade glioma growth. **32**, 491-496 (2018).

2. Heng TS, Painter MW. The Immunological Genome Project: networks of gene expression in immune cells. *Nat Immunol* **9**, 1091-1094 (2008).
